# CoDaLoMic: An R package for modeling microbiome compositional and longitudinal data

**DOI:** 10.1371/journal.pcbi.1014328

**Published:** 2026-06-22

**Authors:** Irene Creus-Martí, Andrés Moya, Francisco J. Santonja

**Affiliations:** 1 Department of Statistics and Operational Research, Universitat de València, Valencia, Spain; 2 Institute for Integrative Systems Biology (I2Sysbio), Universitat de València and CSIC, Valencia, Spain; 3 The Foundation for the Promotion of Health and Biomedical Research of Valencia Region (FISABIO), Valencia, Spain; 4 CIBER in Epidemiology and Public Health (CIBERESP), Madrid, Spain; Universidad de Antioquia, COLOMBIA

## Abstract

In this paper we present *CoDaLoMic*, an R package for analyzing longitudinal and compositional microbiome datasets. The *CoDaLoMic* package implements three models specifically designed for the analysis of microbiome data that are both compositional and longitudinal. Unlike many existing methods that focus solely on pairwise interactions, *CoDaLoMic* also captures interactions among groups of bacteria, providing a more robust methodological framework for studying microbial relationships at the community level. In addition, the package facilitates the analysis of microbiome variability in relation to host health status and allows for the identification of groups of taxa that exhibit similar temporal dynamics. Working with time series data makes it possible to understand not only the current state of a microbial community but also its dynamics over time, which is essential for identifying patterns of ecological succession, detecting events of dysbiosis or recovery, and inferring potential causal relationships between taxa. On the other hand, focusing on interactions among groups of bacteria, rather than analyzing only pairwise relationships, enables a more integrated and functionally meaningful view of the microbiome. Many key ecological functions are the result of the collective behavior of functionally related groups of taxa. Two datasets have been considered in *CoDaLoMic*, one real and one simulated. The real dataset contains the information of the genera present in the microbiome of the *Blatella germanica* cockroach at 105 time points. The simulated dataset is defined taking Lotka-Volterra structure into account. *CoDaLoMic* is available at CRAN.

## Author summary

Understanding how the microbiome changes over time is fundamental to unraveling its role in health and disease, as microbial communities influence processes such as digestion, immune response, and pathogen resistance. In this study, we present *CoDaLoMic*, a user-friendly R package designed to analyze longitudinal and compositional microbiome data, accounting not only for pairwise bacterial interactions but also for collective interactions among functional groups of taxa. This is crucial because many ecological functions emerge from the joint behavior of microbial groups rather than isolated species. *CoDaLoMic* includes advanced models that capture temporal dynamics, enabling the detection of ecological succession patterns, dysbiosis and recovery events, and potential causal relationships among microorganisms. Additionally, the package facilitates the identification of groups of bacteria with similar temporal dynamics, which may reveal shared functions or relevant ecological interactions. We tested *CoDaLoMic* with a real dataset of the gut microbiome of cockroaches across 105 time points, as well as a simulated dataset based on classical ecological models. The package produces publication-ready plots and tables, helping researchers interpret and communicate their biological findings. *CoDaLoMic* is freely available on CRAN and aims to support scientists from various disciplines in the detailed and dynamic study of microbial communities.

## 1. Introduction

The microbiome and health status are related. In fact, techniques are emerging to treat diseases through the microbiome, such as fecal microbiota transplantation [1], probiotic supplementation [1], nutrition-based approaches or the use of the microbiome as a biomarker [2]. In addition, the microbiome has the potential to revolutionize the future of pharmacology due to the interactions between the microbiome and drugs [3]. As a result it is paramount to know more about microbiome dynamics. Microbiota stability over time is related to health status [4]. This fact highlights the importance of studying microbiome time series. The initial approaches to analyzing longitudinal microbiome data included MDSINE [5], which represents an early methodological example in this domain. Table 1 shows the R packages designed to analyse microbiome cross-sectional and longitudinal data. In addition, microbiome data is compositional due to limitations of the techniques used to collect the data [6]. Compositional data carries relative information and for this reason, the compositions are usually expressed in terms of percentages and the sum of the data is subject to a constant constraint [7]. Correlations between compositional data are spurious [8] and as a result, specific methodology is needed to analyze compositional data [9].

**Table 1:** R packages designed to analyze microbiome datasets.

R packages	Aim	Reference
		
*phyloseq*	• Integration of Microbiome Data	[10]
	• Data Wrangling and Transformation	
	• Diversity Analysis	
	• Ordination and Visualization	
	• Differential abundance testing and correlation analysis	
		
*phylogeo*	• Visualizing and Analyzing Phylogeographic Patterns	[11]
		
*MicrobiomeR*	• Data Wrangling and Cleaning	[12]
	• Diversity Analysis	
	• Microbial composition Analysis at different taxonomic levels	
	• Statistical Comparisons	
		
*microbiome*	• Community Composition Analysis	[13]
	• Diversity Analysis	
	• Microbiome Functions and Core Analysis	
	• Transformation and Normalization	
	• Visualization Tools	
		
*microViz*	• Data Visualization	[14]
	• Diversity and Ordination Analysis	
	• Taxonomic Filtering and Transformation	
		
*ggCluster*	• Microbial Networks Analysis	[15]
		
*microbiomeMarker*	• Diversity and Visualization Tools	[16]
	• Diversity and Visualization Tools	
	• Taxonomic Biomarker Identification	
		
*SplinectomeR*	• Handling Longitudinal Data with Irregularities	[17]
	• Visualization and Graphical Analysis	
	• Comparison Between Groups	
	• Group Comparisons	
	• Spline Fitting and Data Smoothing	
	• Permutation-Based Hypothesis Testing	
		
*q2-longitudinal*	• Longitudinal Data Visualization	[18]
	• First Differencing for Temporal Analysis	
	• Paired Sample Analysis	
	• Longitudinal Feature Selection	
	• Differential Abundance Testing Across Time	
		
*BiomeHorizon*	• Longitudinal Data Visualization	[19]
	• Data Compatibility	
	• User-Friendly Interface	
		
*seqtime*	• Microbiome Datasets Simulation	[20]
	• Noise Analysis	
	• Network	
	• Predicting Microbiome Time Series	
*coda4microbiome*	• Application to Cross-sectional and Longitudinal Data	[21]
	• Identification of Microbial Signatures	
	• Graphical Representations	
	• CoDa-Specific Analysis Tools	
		

In the *CoDaLoMic* package, we implemented three models specifically designed to analyze microbiome compositional and longitudinal data, accounting not only for pairwise interactions but also for interactions among groups of bacteria. This approach provides a robust framework for investigating microbial interactions at a group level, extending beyond the pairwise focus of most existing methods. Furthermore, CoDaLoMic facilitates the analysis of microbiome variability in relation to host health status and enables the clustering of taxa that exhibit similar temporal dynamics. However, the package does not support population-level analyses involving comparisons across multiple individuals within a single dataset. In this case, each individual’s time series is modeled independently, although the results can be compared post hoc. *CoDaLoMic* enables multi-subject comparisons across datasets. As presented in [22], our proposal is successful in comparing treated and control microbial populations, revealing distinct behavioral patterns. It also provides insights into the contribution of the entire microbial community to the abundance of individual genera and identifies specific groups of genera that influence particular taxa.

Unlike approaches such as state-space models (SSM) or dynamic Bayesian networks (DBN) the models implemented in *CoDaLoMic*—namely Dirich-gLV, FBM, and BPBM—are specifically formulated within the framework of compositional data, which is methodologically more appropriate for microbiome analysis. These models explicitly employ the Dirichlet distribution and log-ratio transformations such as *alr* or principal balances, thereby respecting the relative structure of the data and avoiding the spurious inferences that may arise when modeling abundances on an absolute scale. Furthermore, the models implemented in *CoDaLoMic* directly capture the temporal self-dependence of each taxon, as well as taxon interactions and community-level effects, through parameters with clear ecological interpretations—such as persistence, competition, or facilitation. The BPBM model, in turn, incorporates Selected Principal Balances (SPBals) to summarize the main ecological gradients of the microbial community, enabling a dimensionality reduction without sacrificing biological interpretability. In contrast, SSMs and DBNs often include latent components or conditional dependency structures which, although powerful from a statistical perspective, can be difficult to interpret ecologically—especially in highly diverse microbiomes with hundreds of taxa. Moreover, these approaches are not always designed to handle compositional data directly, which may require additional transformations or assumptions that are less suitable for this data type. Overall, the models implemented in *CoDaLoMic* offer alternatives that are better aligned with the nature of microbiome data, provide transparent ecological interpretation, and are more practical and scalable for a wide range of applications in the longitudinal analysis of microbial communities. Our proposal offers a unique approach for the analysis of longitudinal microbiome data by combining Bayesian autoregressive modeling with compositional transformations (log-ratios and principal balances), which is not implemented in the other packages reviewed. Unlike tools such as *phyloseq*, *microbiome*, or *microViz*, which are primarily designed for data handling, visualization, and exploratory analysis, CoDaLoMic explicitly models the temporal dynamics of microbial communities while respecting their compositional nature through time-dependent Dirichlet distributions. Compared to packages such as *SplinectomeR* or *q2-longitudinal*, which analyze temporal trajectories without generative modeling, CoDaLoMic enables the inference of causal relationships between groups of taxa (balances) and allows short-term prediction of future community compositions. Even when compared to *coda4microbiome*, which also operates within the log-ratio framework, CoDaLoMic stands out for its ability to estimate microbial interactions through hierarchical Bayesian models and the use of principal balances, thus reducing dimensionality while maintaining biological interpretability. In summary, CoDaLoMic complements the existing ecosystem of microbiome tools by providing a robust framework for compositional, longitudinal, and inferential modeling.

## 2. Design and Implementation

### 2.1. Data

This R package contains two datasets, one simulated and one real. The simulated dataset is called Simulated and the real dataset is called cockroach. Simulated is a small dataset with 5 microbial taxa and 10 time points, designed to develop tests with models for microbiota without large computational requirements. The major dataset in the package is the real dataset, with 105 time points and 210 genera.

For the simulated dataset, the interaction matrix is constructed using the algorithm proposed by Klemm and Eguíluz in [23], which generates scale-free networks with small-world properties. In this context, each node represents a species, and edges denote potential ecological interactions. The algorithm builds the network incrementally by connecting new nodes to a dynamically maintained set of active nodes, favoring recently added species while allowing random long-range connections. The resulting binary incidence matrix encodes the presence or absence of interactions between species and serves as the structural backbone for assigning interaction strengths in ecological models such as generalized Lotka-Volterra dynamics. Following the scheme given by [24], we generated the interaction matrix using the algorithm proposed by K. Klemm and V. M. Eguluz in [23] and we generated the initial abundances using the Poisson distribution. Taking both of them into account, we simulated the data using the generalized Lotka–Volterra structure. We carried out the simulation using the R package seqtime [24]. Note that this package is designed for the analysis of sequencing data over time and for simulating community dynamics. Focusing on technical details, to generate the interaction matrix we set the clique size at 4, the diagonal values at -1, the interaction connectance at 0.04, the positive edge percentage at 64%, and the maximal absolute off-diagonal interaction strength at 1. Note that we consider four species (or nodes) where every member interacts with every other member. Setting diagonal values to -1 means each species has a negative self-effect, which prevents unbounded growth. Connectance is the proportion of nonzero interactions in the matrix. A 64% positive edge percentage means that 64% of the interactions are positive (beneficial), and the rest (36%) are negative. Off-diagonal elements represent interactions between different species. The quality of the estimation for the Dirich-gLV, FBM and BPBM models using this dataset can be observed in S1–S3 Tables.

The real data is extracted from [25], more specifically, the data is the information on the K3 cockroach in the article [25]. This dataset contains 105 time points and 210 genera. It is a gut microbiome dataset of a *B. germanica* cockroach treated by kanamycin during three periods of time (days: 1–10, 36–45, 71–80). The quality of the estimation for the Dirich-gLV, FBM and BPBM models using this dataset can be observed in S4–S8 Tables.

### 2.2. Availability of code, data and code tutorials

CoDaLoMic is an R package available on CRAN (https://CRAN.R-project.org/package=CoDaLoMic). The datasets employed in this study (both simulated and the cockroach [25] dataset) are included within the CoDaLoMic package.

Additionally, the GitHub repository CoDaLoMic-Tutorial (https://github.com/Creus-Marti/CoDaLoMic-Tutorial.git) provides a detail explanation of the models implemented, the scripts used to generate the results presented in this paper and tutorials illustrating how to analyze data using CoDaLoMic. In addition, both the GitHub repository and the S1 Appendix provide a detailed explanation and a pipeline of the preprocessing stages.

### 2.3. Model selection criteria

CoDaLoMic provides implementations of three models: Dirich-gLV [26], FBM [27], and BPBM [28]. A detailed description of these models is available in the GitHub repository (https://github.com/Creus-Marti/CoDaLoMic-Tutorial.git) and in S2 Appendix. Table 2 summarizes the key considerations for selecting one model over another, in relation to the specific objectives intended to be achieved through its application.

**Table 2:** Comparison of microbial community models based on biological relevance and practical considerations.

Model	Biological Focus	Strengths and Limitations	Ideal Applications
Dirich-gLV	Captures detailed pairwise interactions and self-influence of taxa, useful for understanding direct ecological relationships.	Strength: Explicit modeling of taxon-taxon effects. Limitation: Parameter explosion as taxa increase, making it less scalable for large communities.	Moderate-sized communities where detailed interaction dynamics are crucial, e.g., targeted ecological studies.
FBM	Balances self-dependence with community-wide compositional influence through aggregated balances.	Strength: Captures both taxon inertia and community effects. Limitation: Less granular pairwise interaction detail.	Communities where taxa are influenced by overall community shifts rather than specific pairwise relations.
BPBM	Focuses on large-scale community structure via Selected Principal Balances representing dominant ecological gradients.	Strength: Dimension reduction enabling scalability and interpretability. Limitation: May obscure fine-scale interactions between individual taxa.	Highly diverse microbiomes with complex data where understanding broad ecological patterns is prioritized.

**Fig 1:**
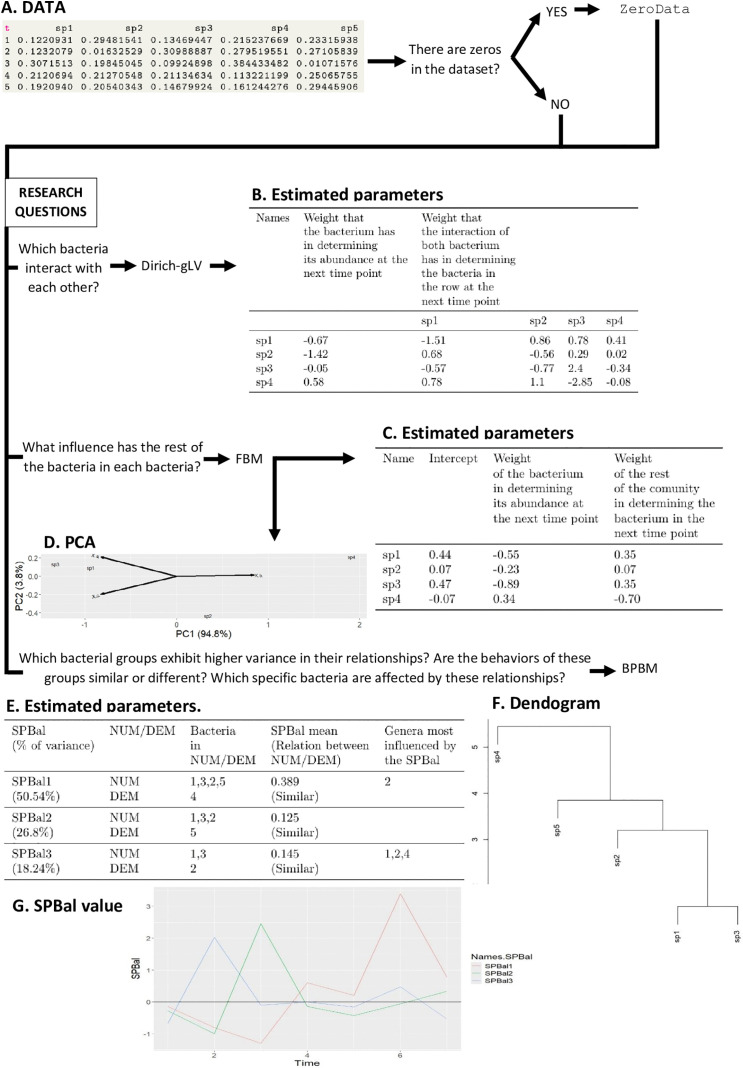
Describing microbiome dynamics. *CoDaLoMic* is able to describe microbiome dynamics. A: Data structure. B: Estimated parameters obtained with the Dirich-gLV model. C: Estimated parameters obtained with the FBM model. D: PCA of the estimated parameters (obtained with FBM model). E: Estimated parameters obtained with the BPBM model. F: Dendogram with the principal balances. G: Value of the Selected Principal Balances (SPBal) during all time points. Panels B, C and E are also available (in LATEX format) in S1 Text.

**Fig 2:**
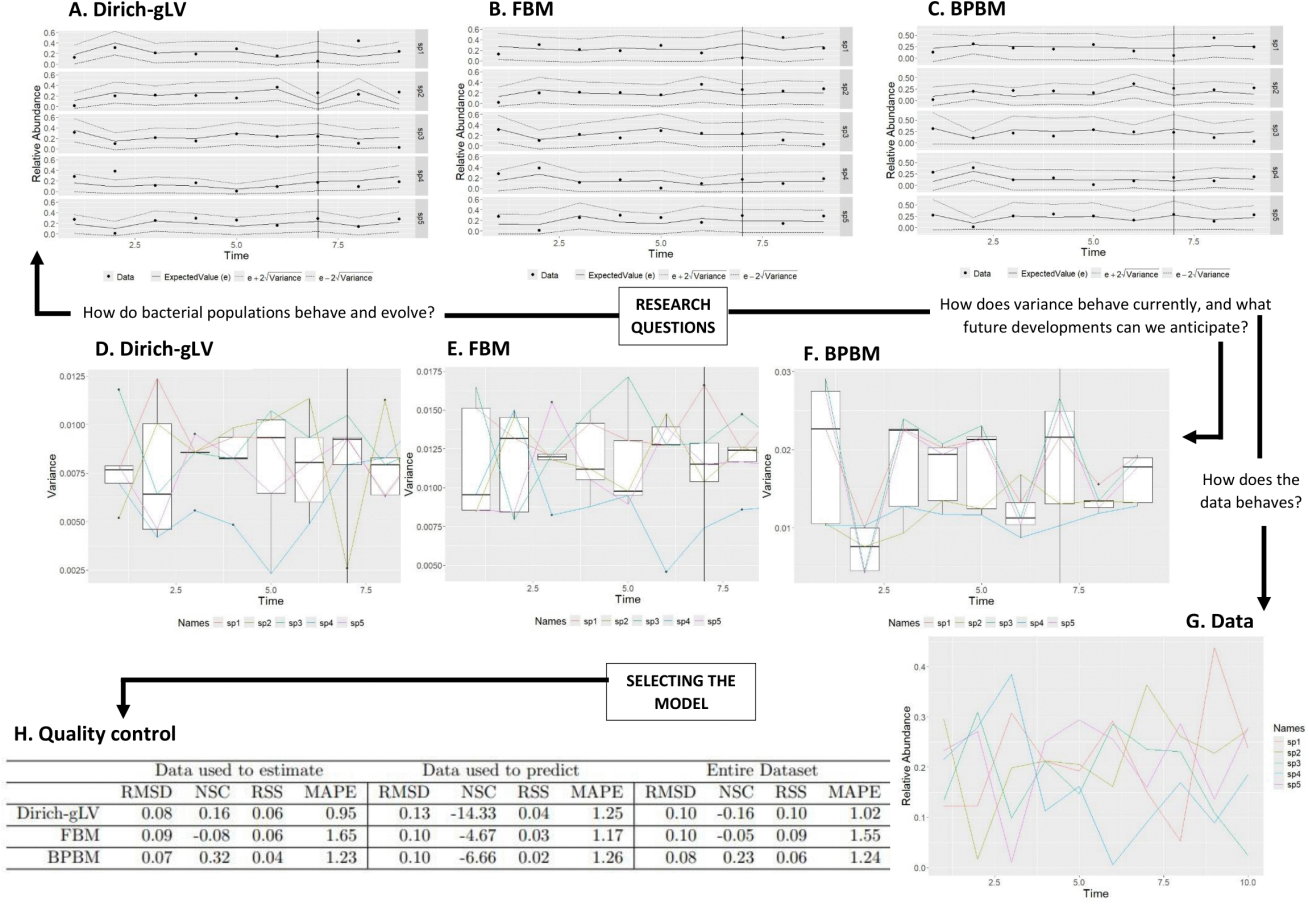
Describing and predicting microbiome dynamics. *CoDaLoMic* describes and predicts microbiome dynamics. Panels A, B, and C show the expected values generated by each model; panels D, E, and F display the corresponding variances. The vertical line indicates the point at which prediction begins. Panel G presents a graphical overview of the dataset, which is identical across all three models. Panel H shows the mean value of the RMSD, NSC, RSS and MAPE for the three models. Panel H is also available in LATEX format in S1 Text.

For further detail, Figs 1 and 2 illustrate the research questions addressed by each model and the corresponding outputs generated to answer them. Specifically, Fig 1 presents the outputs and questions related to dataset description while Fig 2 extends this by including both descriptive and predictive analyses. Interpretation of the output is explained in the model tutorials available in the GitHub repository (https://github.com/Creus-Marti/CoDaLoMic-Tutorial.git).

Note that part A in Fig 1 illustrates the structure of the input data required by the package; part B, C and E in Fig 1 interpret the estimated parameters obtained for each model; and part D in Fig 1 presents the estimated parameters of the FBM model in a graphical format. Fig 1 appears the concept of Principal Balances and Selected Principal Balances (SPBal), they denote a log-ratio contrast between groups of taxa selected for their capacity to account for the majority of variance in compositional data. A higher absolute value of the SPBal indicates a greater difference between the groups. The SPBal are the PB for which the sum of the percentage of variance is higher than 80%. More detail can be found in S2 Appendix or in the GitHub repository (https://github.com/Creus-Marti/CoDaLoMic-Tutorial.git). As a result, part F in Fig 1 shows a dendrogram that groups together the taxa whose relationships maximize the variance and part G in Fig 1 shows the degree of similarity or dissimilarity between the groups that maximize the variance across all time points. In addition, part A, B and C in Fig 2 show the expected values generated by each model, serving as a graphical assessment of model fit; part D, E and F in Fig 2 present the variance analysis; part G in Fig 2 describes the dataset; and part H in Fig 2 displays the quality indices.

In summary, the choice of model depends on the specific analytical focus: Dirich-gLV is most appropriate when investigating pairwise interactions; FBM should be selected when both taxa-level and community-level effects are of interest; and BPBM is recommended for analyses centered on community structure. All three methods require that the time sampling frequency be equidistant across all time points. This interval may be daily, weekly, monthly or any other consistent temporal lag, but the distance between consecutive time points must remain uniform. If any time point is missing, imputation methods must be employed for estimation.

## 3. Results

In this section, a comparative performance analysis of CoDaLoMic will be conducted against all methods presented in Table 1 that are specifically designed for longitudinal data. These methods include: q2-longitudinal, coda4microbiome, SplinectomeR, BiomeHorizon and seqtime. Table 3 shows the results of the comparion.

**Table 3:** Comparison between the proposed methods and existing approaches across various aspects: input characteristics, research questions addressed and computational characteristics and performance. Only two methods accept the same input type (Seqtime and BiomeHorizon). These are compared methodologically based on the types of problems they address. Computational characteristics and performance are evaluated against Seqtime, as BiomeHorizon is designed for visualization rather than dynamic estimation and does not support RMSD calculation. The table reports RMSD for estimation and prediction, as well as computation time equired to estimate the model.’tp’ denotes time point, “s” seconds, “d” days, ‘–’ indicates model failure and * indicates models implemented in CoDaLoMic.

			q2-longitudinal	coda4microbiome	SplinectomeR	BiomeHorizon	Dirich-gLV*	FBM*	BPBM*	seqtime
		Multiple individuals	✓	✓	✓	✓				
		An individual				✓	✓	✓	✓	✓
	Microbial	Time points equally spaced					✓	✓	✓	✓
Input Characteristics	abundance	Time points not equally spaced	✓	✓	✓	✓				
	over time	Percentage data		✓	✓	✓	✓	✓	✓	✓
		Count data	✓	✓	✓	✓				✓
	Information distinguishing individuals by at least one characteristic		✓	✓	✓					
	Simulate microbiome datasets									✓
	Noise analysis									✓
	Pair-wise interaction						✓			✓
	Network									✓
	Predicting microbiome time series						✓	✓	✓	✓
	Microbiome visualization					✓	✓	✓	✓	✓
Research	Compare abundant and rare taxa concurrently					✓				
Questions	Influence of individual and collective bacteria on overall community composition							✓		
	Identify bacterial groups with high relational variance								✓	
	Characterize interaction patterns between groups with high relational variance								✓	
	Identify bacteria shaped by high-variance group interactions								✓	
	Current and future variance						✓	✓	✓	
	Dataset	Quality metric	Dirich-gLV		FBM		BPBM		seqtime	
	10 taxa	RMSD (describe)	0.150		0.053		0.052		0.081	
	100 tp	RMSD (predict)	–		0.055		0.055		0.073	
	20 tp predicted	Computational time	1943.411s		1211.178s		8332.643s		1.692s	
	20 taxa	RMSD (describe)	–		0.028		0.027		0.042	
Computational	100 tp	RMSD (predict)	–		0.030		0.027		0.043	
Characteristics	20 tp predicted	Computational time	–		3439.741s		7916.412s		17.075s	
and	40 taxa	RMSE (describe)	–		0.017		0.015		0.024	
Performance	100 tp	RMSD (predict)	–		0.018		0.016		0.025	
	20 tp predicted	Computational time	–		8810.686s		8.866d		137.870s	
	60 taxa	RMSD (describe)	–		0.013				–	
	100 tp	RMSD (predict)	–		0.021				–	
	20 tp predicted	Computational time	–		15534.914s		> 15*d*		–	
	80 taxa	RMSD (describe)	–		0.008				–	
	100 tp	RMSD (predict)	–		0.011				–	
	20 tp predicted	Computational time	–		4713.127s		> 15*d*		–	

First, Table 3 details the characteristics of the input data required by the different methods. Notably, q2-longitudinal, coda4microbiome and SplinectomeR are suitable for analyzing multiple individuals and necessitate metadata to distinguish between subjects. Conversely, CoDaLoMic, BiomeHorizon, and seqtime are not designed for this type of input data. Consequently, a direct comparison of the performance of CoDaLoMic with q2-longitudinal, coda4microbiome and SplinectomeR is not feasible, as these methods address fundamentally different research questions.

Secondly, Table 3 illustrates the distinctions among the research questions addressed by CoDaLoMic, BiomeHorizon and seqtime. We can observe that several research questions are addressed exclusively by CoDaLoMic, which immediately demonstrates the utility of CoDaLoMic regardless of the other proposed methods. Thirdly, we will compare the performance and computational efficiency of CoDaLoMic and seqtime in describing and predicting microbiome dynamics across datasets varying in the number of taxa. Notably, BiomeHorizon is excluded from this comparison, as its primary design objective is visualization rather than dynamic description or prediction.

Table 3 presents the Root Mean Square Deviation (RMSD) values calculated for both the descriptive and predictive capacity of the different methods in datasets of different lengths. Additionally, the table details the computational time required for the estimation process across datasets of varying sizes. The compilations were performed on an HPC cluster running Rocky Linux 8.10, consisting of 13 compute nodes with a total of 608 CPU cores (1216 threads with hyper-threading) and 15 TB of RAM. The system is equipped with a distributed Ceph storage architecture incorporating SSD/NVMe drives and an internal network bandwidth of 20 Gb/s. The peak performance of the cluster, as measured by HPLinpack 2.3, is 7 Tflops/s. In order to simulate the datasets of different lengths, we first constructed the interaction matrix by drawing its entries randomly from a uniform distribution [23]. We then generated the initial bacterial abundances using a Poisson distribution [24]. Combining these components, we simulated the community dynamics under a generalized Lotka–Volterra framework. All simulations were performed using the R package seqtime [24].

Table 3 shows that BPBM achieves the highest accuracy in both description and prediction, although it is also the most computationally demanding method. FBM attains results comparable to BPBM while requiring substantially less computation time. Seqtime is the fastest approach, but it yields the lowest accuracy. In addition, seqtime is unable to produce results for the largest datasets because, during the prediction step, it is not possible to solve de Lotka Volterra equations. A similar issue arises with the Dirichlet‑gLV model, it often fails to produce stable results because it is highly sensitive to atypical values. In estimating the Dirichlet parameter, an exponential transformation is applied; however, this transformation may diverge to infinity, which prevents the computation of the predicted values. This sensitivity to outliers can also hinder the estimation of the expected values, not only the predicted ones.

We will now address the comparison of research questions using a case study based on the cockroach dataset (see a description of the dataset in part A of S1 Fig), with particular emphasis on interpreting the outputs of CoDaLoMic. Part A in Table 4 indicates that the Dirich-gLV model provides the poorest fit to the cockroach data, as it exhibits the highest values for RMSD, RSS and MAPE. Furthermore, its NSC value is the furthest from one. Seqtime improves upon Dirich-gLV, however, its performance is further enhanced by FBM and BPBM. FBM and BPBM produce similar results, but BPBM enhances all values, except for RMSD, which remains the same for both models. Note that BiomeHorizon is designed for visualization rather than calculation and do not support the calculation of these indexes. Considering this information, we will analyze the results obtained with seqtime (see part A, B, C and D of S2 Fig), BiomeHorizon (see S3 Fig), FBM and BPBM when estimating the cockroach dataset.

**Table 4:** Table with the information of the cockroach dataset. A: mean values of RMSD, NSC, RSS and MAPE for all models. B: Parameter information and its interpretation when using FBM. C: Information and interpretation obtained with the estimation of BPBM, we assign a number to each bacterium for easier identification: 1 is g_Dysgonomonas, 2 is g_Bacteroides, 3 is f_Lachnospiraceae, 4 is g_Desulfovibrio, 5 is g_Candidatus_Soleaferrea, 6 is g_Alistipes, 7 is f_Ruminococcaceae, 8 is c_Bacteroidia, 9 is g_Breznakia, 10 is f_Tannerellaceae, 11 is g_Christensenellaceae_R-7_group, 12 is f_Dysgonomonadaceae, 13 is c_vadinHA49, 14 is g_Desulfatiferula, 15 is Other.

A. Model	RMSD	NSC	RSS	MAPE
Dirich-gLV	0.22	-272.82	6.48	3.22
FBM	0.03	-1.17	0.16	1.69
BPBM	0.03	-0.89	0.12	1.41
seqtime	0.05	-2.22	0.46	1.69
**B. FBM: Estimated Parameters**				
**Bacteria**	**Intercept**	**Weight (bacteria)**	**Weight (comunity)**	
g_Dysgonomonas	0.9142	0.1210	0.4037	
g_Bacteroides	-0.3339	0.1635	0.4250	
f_Lachnospiraceae	-0.2854	0.6590	0.1235	
g_Desulfovibrio	-0.0563	0.2611	0.1830	
g_Candidatus_Soleaferrea	0.7683	0.5984	0.5220	
g_Alistipes	-0.0169	0.3628	0.2926	
f_Ruminococcaceae	0.6922	0.3463	0.6383	
c_Bacteroidia	-0.3684	0.4558	0.2647	
g_Breznakia	-1.6318	0.1285	0.4090	
f_Tannerellaceae	-0.0061	0.5205	0.3316	
g_Christensenellaceae_R-7_group	-1.6030	0.1806	0.0921	
f_Dysgonomonadaceae	-1.1354	0.1023	0.5145	
c_vadinHA49	-1.8314	0.1053	0.3434	
g_Desulfatiferula	-1.0554	0.3422	0.1563	
**C. BPBM: SPBal and interpretation**				
**SPBal**	**Bacteria in NUM/DEM**	**Media SPBal**	**Most Influenced Genera**	
**(% of variance)**		**(Relationship)**		
SPBal1	NUM: 13,14,9	-0.531	1,4,5,6,10,11,15	
(17.74%)	DEM: 12	(Similar)		
SPBal2	NUM: 13,14	0.01	1,4,7	
(12.35%)	DEM: 9	(Similar)		
SPBal3	NUM: 8,10,3	0.363	1,2,3,5,6,8,10,11,13,15	
(10.84%)	DEM: 11	(Similar)		
SPBal4	NUM: 5,7,6	0.301	1,4,7	
(9.59%)	DEM: 2	(Similar)		
SPBal5	NUM: 13,14,9,12	-3.983	1,2,4,15	
(9.53%)	DEM: 1,15,4,5,7,6,2,8,10,3,11	(Different)		
SPBal6	NUM: 8,10	0.061	1,2,3,4,5,6,8,9,10,11	
(7.68%)	DEM: 3	(Similar)		
SPBal7	NUM: 13	-0.209	1,4,7,11,12	
(6.09%)	DEM: 14	(Similar)		
SPBal8	NUM: 8	-0.007	1,2,4,10,15	
(5.66%)	DEM: 10	(Similar)		
SPBal9	NUM: 5,7,6,2	1.3	1,3,4,5,6,7,8,11,15	
(5.62%)	DEM: 8,10,3,11	(Different)		

Part B in Table 4 taxa versus community dynamics as explained by FBM. For the taxa g__Dysgonomonas, g__Bacteroides, f__Ruminococcaceae, g__Breznakia, f__Dysgonomonadaceae, and c__vadinHA49, the community weight is higher than the self-weight in defining their abundance at the subsequent time point (we call them group C). Conversely, g__Candidatus_Soleaferrea is unique in exhibiting similar influence from both the bacterium itself and the rest of the community. For all remaining bacteria, the self-influence predominates over the community influence in determining future abundance (we call them group B). These dynamics are also observable in the PCA present in part B of S1 Fig, where groups C and B exhibit clear separation.

Part C in Table 4 details the inter-taxa dynamics explained by BPBM. Within Group B, 8,10,3 (c_Bacteroidia, f_Tannerellaceae, f_Lachnospiraceae) and 11 (g_Christensenellaceae_R-7_group) exhibit similar behavior, and their combined relationship exerts the primary influence across the majority of the dataset.Taxa within Group C interact cooperatively with Group B members. Specifically, 13,14,9 (c_vadinHA49, g_Desulfatiferula, g_Breznakia) and 12 (f_Dysgonomonadaceae) share similar behavior and chiefly influence the bacteria in Group B. Furthermore, the combined set of taxa 5,7,6,2 (g_Candidatus_Soleaferrea, f_Ruminococcaceae, g_Alistipes, g_Dysgonomonas) and 8,10,3,11 (c_Bacteroidia, f_Tannerellaceae, f_Lachnospiraceae, g_Christensenellaceae_R-7_group) display divergent behavior, with their relationships collectively impacting nearly the entire dataset. Finally, the collection of taxa 13,14,9,12 (c_vadinHA49, g_Desulfatiferula, g_Breznakia, f_Dysgonomonadaceae) exhibits behavior distinct from the rest of the dataset (a separation also depicted in part B of S4 Fig) and its interaction primarily influences genera 1,2,4,15 (g__Dysgonomonas, g__Bacteroides, g__Desulfovibrio, Other).

It must be noted that S5 and S6 Figs presents the expected values obtained from the FBM and BPBM models, and visually confirms the goodness-of-fit of the models to the observed data. Before concluding, note that both the BPBM and FBM methods allow variance analysis (see part C of S1 Fig and part C of S4 Fig) and that BPBM additionally produces a dendogram showing the groups of bacteria whose relationships exhibit the maximum variability (see part A of S4 Fig).

In S2 Fig we observe the network visualization provided by seqtime. Specifically, we observe a closed-loop dynamic wherein taxon 1 benefits from taxon 2, which in turn is sequentially benefited by taxa 11, 7, 5, 9, 13, and 10, completing the cycle back to taxon 1.Conversely, S3 Fig presents the results obtained using BiomeHorizon. It is observed that while most taxa exhibit alternating periods of high and low abundance, taxa 2, 14, and 15 display a distinct pattern: they are highly abundant initially but subsequently experience a marked decrease in abundance.

The analysis of the cockroach dataset, utilizing the various methods, unequivocally demonstrates that each approach is tailored to address distinct research objectives. The innovation inherent in the FBM and BPBM lies specifically in their capacity to describe microbiome dynamics taking into account the relationships between groups of bacteria.

## 4. Availability and future directions

A deeper understanding of microbiome dynamics is crucial, as microbial stability over time is directly associated with host health status [4]. This necessitates the study of microbiome time series to facilitate the development of effective, mechanism-based clinical treatments.

*CoDaLoMic* is a comprehensive R package (available on CRAN: https://CRAN.R-project.org/package=CoDaLoMic)designed to model and predict the dynamics of microbiome communities over time. It leverages advanced models such as Dirich-gLV, FBM, and BPBM to examine microbial abundance and the intricate relationships between various bacterial groups at different time points. What sets *CoDaLoMic* apart is its ability to predict future microbial abundances based on the interactions between bacterial taxa, providing a more nuanced understanding of how changes in one group can directly or indirectly affect others. The package allows for the estimation of these relationships using maximum likelihood estimation (for Dirich-gLV and FBM models) and MCMC (for BPBM), enabling researchers to analyze complex microbiome data over extended periods.

The focus of *CoDaLoMic* on prediction and its capacity to model microbial interactions over time makes it particularly valuable for longitudinal microbiome studies. It addresses the challenge of understanding not only the current state of the microbiome but also how it will evolve based on existing patterns and relationships. In contrast to other R packages, such as *coda4microbiome*, *q2-longitudinal*, or *SplinectomeR*, which focus on different aspects of microbiome analysis—such as community composition, diversity metrics, or statistical associations—*CoDaLoMic* goes beyond just descriptive analysis. It emphasizes predictive modeling, making it especially useful for research aiming to understand the long-term dynamics of microbiome populations, such as in studies of health interventions, environmental changes, or disease progression.

Additionally, *CoDaLoMic* incorporates the concept of principal balances (SPBal), offering a unique approach capturing the interactions between bacterial groups and their collective influence on microbiome composition. This allows for a more precise and actionable understanding of microbial community behavior and has the potential to inform clinical or therapeutic decisions based on microbiome data. The package also provides detailed outputs in LaTeX format, facilitating the inclusion of results in scientific publications.

However, the package also has limitations. As the size of the dataset increases, the computation time required for model estimation grows significantly. In cases where the dataset is particularly large, these computational demands may exceed the capacity of standard desktop computers, necessitating the use of more powerful external servers or cloud-based systems to perform the necessary calculations efficiently. This issue is especially relevant for large-scale microbiome studies where computational resources can become a bottleneck. Furthermore, the current models implemented in *CoDaLoMic* are primarily designed to analyze the microbiome dynamics of a single subject at a time. The package does not currently support population-level analyses involving simultaneous modeling across multiple individuals within a single dataset. Instead, each individual’s time series is modeled independently, with results compared post hoc. While *CoDaLoMic* enables multi-subject comparisons across separate datasets, integrated multi-subject modeling within the same dataset remains a future development goal. Additionally, the current models do not yet incorporate external covariates such as dietary factors, host health status, medication use, or other environmental variables. These factors, together with batch effects, are well-recognized sources of variability in microbiome studies and can significantly influence microbial composition and dynamics. Therefore, it is crucial to apply appropriate preprocessing corrections prior to using *CoDaLoMic* to avoid confounding effects. We recommend performing batch correction on data transformed by log-ratio methods (e.g., clr or alr transformations) to preserve the intrinsic compositional structure of the data. In contrast, preprocessing techniques such as rarefaction or total sum scaling normalization can distort compositional relationships and compromise the validity of downstream modeling results; thus, these approaches are discouraged. Using *CoDaLoMic*, subjects should be analyzed on an individual basis, enabling post hoc analysis to explore associations between the longitudinal data behavior and health status, treatment type, or other relevant clinical variables. Looking forward, future versions of *CoDaLoMic* will explicitly incorporate external covariates and batch effects within the modeling framework. This enhancement will enable more direct and robust analyses of their impact on microbiome dynamics, particularly benefiting clinical or dietary intervention studies by improving the models’ ability to capture complex, context-dependent microbial community behaviors. Integrating these covariates is expected to improve both the accuracy and interpretability of predictions derived from longitudinal microbiome data. Looking ahead, there are several key areas for future development. A major objective is to extend the models to handle data from multiple subjects simultaneously within the same dataset, enabling more comprehensive population-level analyses. This advancement will facilitate comparative studies across individuals or groups exposed to different treatments or environmental factors. Additionally, ongoing optimization of the code will focus on improving computational efficiency and reducing runtime, ensuring the package remains scalable and efficient when applied to very large datasets.

## Supporting information

S1 Table Simulated dataset. **Dirich-gLV. Estimation quality.** Value of the parameters in the last iterations of the optimization procedure to obtain the maximum likelihood estimation. The names of the parameters follow the notation in Equation 3. We can see that the values are identical, indicating that the optimization procedure has converged.
(PDF)

S2 Table Simulated dataset. **FBM. Estimation quality.** Value of the parameters in the last iterations of the optimization procedure to obtain the maximum likelihood estimation. The names of the parameters follow the notation in Equation 4. We can see that the values are identical, indicating that the optimization procedure has converged.
(PDF)

S3 Table Simulated dataset. **BPBM. Estimation quality.** Parameter information after obtaining the parameters of the BPBM model using MCMC. The first two columns represent the parameter names as described in Equation 5 and the corresponding names of the parameters outputted by R, respectively. The parameters that have a mean of zero, but non-zero values for the standard deviation and quantiles, are those whose credible intervals include zero at the center. The StudyingParam function has set their mean to zero. Since the estimated Rhat is less than 1.1 and the effective sample size (n.eff) exceeds 100, the quality of the estimation can be considered satisfactory.
(PDF)

S4 Table Cockroach dataset. **Dirich-gLV. Estimation quality.** Parameter values from the final iterations of the optimization procedure to obtain the maximum likelihood estimation. Due to the high quantity of parameters, the information for all the parameters is in two tables, S4 and S5 Tables. We can see that the values are identical, indicating that the optimization procedure has converged.
(PDF)

S5 Table Cockroach dataset. **Dirich-gLV. Estimation quality.** Parameter values from the final iterations of the optimization procedure to obtain the maximum likelihood estimation. Due to the high quantity of parameters, the information for all the parameters is in two tables, S4 and S5 Tables. We can see that the values are identical, indicating that the optimization procedure has converged.
(PDF)

S6 Table Cockroach dataset. **FBM. Estimation quality.** Parameter values from the final iterations of the optimization procedure to obtain the maximum likelihood estimation. The parameters are named according to the notation in Equation 4. Since the values are the same, it indicates that the optimization procedure has converged.
(PDF)

S7 Table Cockroach dataset. **BPBM. Estimation quality.** Parameter information after obtaining the parameters of the BPBM model using MCMC. Due to the high quantity of parameters, the information for all the parameters is in two tables, S7 and S8 Tables. The parameters with a mean of zero, but non-zero values for the standard deviation and quantiles, are those whose credible intervals include zero at the center. The StudyingParam function has adjusted their mean to zero. Since the estimated Rhat is less than 1.1 and the effective sample size (n.eff) exceeds 100, the quality of the estimation can be considered satisfactory.
(PDF)

S8 Table Cockroach dataset. **BPBM. Estimation quality.** Parameter information after obtaining the parameters of the BPBM model using MCMC. Due to the high quantity of parameters, the information for all the parameters is in two tables, S7 and S8 Tables. The parameters with a mean of zero, but non-zero values for the standard deviation and quantiles, are those whose credible intervals include zero at the center. The StudyingParam function has adjusted their mean to zero. Since the estimated Rhat is less than 1.1 and the effective sample size (n.eff) exceeds 100, the quality of the estimation can be considered satisfactory.
(PDF)

S1 Fig Results obtained with FBM in cockroach dataset. A. Temporal representation of taxa across all time points. B. Principal Component Analysis (PCA) of the estimated parameters, enabling visualization of bacterial taxa with similar dynamics; taxa positioned closer together in the PCA space exhibit more similar behavior. C. Variance over time.
(PDF)

S2 FigSeqtime.Results obtained using seqtime in cockroach dataset. In panels A and D, red indicates negative interactions while green denotes positive ones. Panel B reveals that the simulated correlation is not higher than the lag-1 autocorrelation, suggesting that the interaction matrix contributes little beyond the inherent temporal inertia of the data. We assign a number to each bacterium for easier identification: 1 is g_Dysgonomonas, 2 is g_Bacteroides, 3 is f_Lachnospiraceae, 4 is g_Desulfovibrio, 5 is g_Candidatus_Soleaferrea, 6 is g_Alistipes, 7 is f_Ruminococcaceae, 8 is c_Bacteroidia, 9 is g_Breznakia, 10 is f_Tannerellaceae, 11 is g_Christensenellaceae_R-7_group, 12 is f_Dysgonomonadaceae, 13 is c_vadinHA49, 14 is g_Desulfatiferula, 15 is Other.(PDF)

S3 Fig BiomeHorizon. Results obtained with BiomeHorizon in cockroach dataset. Values are centered around a reference point. The plotting area is segmented into quartile bands extending above and below this origin. Darker blue bands represent progressively higher values above the origin, while darker red bands indicate increasingly lower values below it. Negative bands are symmetrically mirrored upward to enhance visual interpretation.
(PDF)

S4 Fig Results obtained with BPBM in cockroach dataset. A: Dendrogram illustrating the Principal Balances. B: Temporal profile of the selected Principal Balances across all time points. Values closer to zero indicate greater similarity in the relationships between the groups within each balance. C: Variance of the taxa over time.
(PDF)

S5 FigExpected values obtained with the FBM model.The abbreviation Dys stands for g_Dysgonomonas, while Bct represents g_Bacteroides. These are followed by Lac, which corresponds to f_Lachnospiraceae, and Dsf, which refers to g_Desulfovibrio. Continuing on, Can is the abbreviation for g_Candidatus_Soleaferrea, and Ali represents g_Alistipes. Rum corresponds to f_Ruminococcaceae, whereas Bac refers to c_Bacteroidia. In the next set, Brz stands for g_Breznakia, and Tan refers to f_Tannerellaceae. Meanwhile, Chr represents g_Christensenellaceae_R7_group, and Dgn stands for f_Dysgonomonadaceae. Lastly, Vad corresponds to c_vadinHA49, and Dfa represents g_Desulfatiferula. The abbreviation Oth simply refers to Other.(PDF)

S6 FigExpected values obtained with the BPBM model.The abbreviation Dys stands for g_Dysgonomonas, while Bct represents g_Bacteroides. These are followed by Lac, which corresponds to f_Lachnospiraceae, and Dsf, which refers to g_Desulfovibrio. Continuing on, Can is the abbreviation for g_Candidatus_Soleaferrea, and Ali represents g_Alistipes. Rum corresponds to f_Ruminococcaceae, whereas Bac refers to c_Bacteroidia. In the next set, Brz stands for g_Breznakia, and Tan refers to f_Tannerellaceae. Meanwhile, Chr represents g_Christensenellaceae_R7_group, and Dgn stands for f_Dysgonomonadaceae. Lastly, Vad corresponds to c_vadinHA49, and Dfa represents g_Desulfatiferula. The abbreviation Oth simply refers to Other.(PDF)

S1 Appendix Preprocessing, quality control, zero imputation and impact on modeling. A document that includes a detailed explanation and a pipeline of the preprocessing stage.
(PDF)

S2 Appendix Models implemented in CoDaLoMic. Document detailing the three models implemented in CoDaLoMic (Dirich-gLV, FBM, and BPBM).
(PDF)

S1 Text Supporting information. A document that includes the tables included in Figs 1 and 2 in an editable, cell‑based LATEX format.
(PDF)
