# Supplementary material for: CoDaLoMic: An R package for modeling microbiome compositional and longitudinal data
Source: PLoS Comput Biol. 2026 Jun 22;22(6):e1014328. doi: 10.1371/journal.pcbi.1014328 (PMC13362355; doi:10.1371/journal.pcbi.1014328)
Supplement: S1 Table — Dirich-gLV. Estimation quality. Value of the parameters in the last iterations of the optimization procedure to obtain the maximum likelihood estimation. The names of the parameters follow the notation in Equation 3. We can see that the values are identical, indicating that the optimization procedure has converged. (PDF) [file pcbi.1014328.s001.pdf]

Table S1. Simulated dataset. Dirich-gLV. Estimation quality. Value of the parameters in the last iterations of the optimization procedure to obtain the maximum likelihood estimation. The names of the parameters follow the notation in Equation 3. We can see that the values are identical, indicating that the optimization procedure has converged.

Parameter	τ	r_1	r_2	r_3	r_4	a_{11}
iteration495	18.47606	-0.6732475	-1.428132	-0.04412525	0.5893751	-1.505913
iteration496	18.48665	-0.6734658	-1.427472	-0.0430771	0.5892333	-1.506301
iteration497	18.37922	-0.663682	-1.425697	-0.04306986	0.5910236	-1.510869
iteration498	18.52041	-0.6688477	-1.425567	-0.04247036	0.5845098	-1.512716
iteration499	18.44622	-0.6698447	-1.423804	-0.03987477	0.5864225	-1.512789
iteration500	18.41739	-0.6727171	-1.423634	-0.05198025	0.5841157	-1.512707

Parameter	a_{21}	a_{31}	a_{41}	a_{12}	a_{22}	a_{32}
iteration495	0.6652498	-0.5557873	0.7744096	0.8553246	-0.5549019	-0.7831572
iteration496	0.666549	-0.5564218	0.7744137	0.8554262	-0.554908	-0.7837468
iteration497	0.6718005	-0.5628366	0.7720237	0.8548694	-0.5587882	-0.7801501
iteration498	0.6752144	-0.5650099	0.7800802	0.8600821	-0.5578617	-0.7743886
iteration499	0.6791762	-0.5698769	0.7782153	0.8573007	-0.5590324	-0.775067
iteration500	0.6778131	-0.5677674	0.7819926	0.8564527	-0.5580653	-0.772963

Parameter	a_{42}	a_{13}	a_{23}	a_{33}	a_{43}	a_{14}
iteration495	1.113716	0.76042	0.304136	2.400481	-2.852168	0.4168907
iteration496	1.112194	0.7617065	0.3028321	2.402811	-2.851114	0.4167121
iteration497	1.111498	0.7674838	0.3008907	2.408839	-2.847346	0.4152484
iteration498	1.106172	0.7685285	0.294204	2.404059	-2.851379	0.412919
iteration499	1.105984	0.7758394	0.2897033	2.405429	-2.846494	0.4129016
iteration500	1.104241	0.777381	0.2870295	2.403598	-2.850048	0.4123578

Parameter	a_{24}	a_{34}	a_{44}
iteration495	0.02355565	-0.3399439	-0.07851503
iteration496	0.02384059	-0.3398575	-0.07822873
iteration497	0.02393027	-0.3389321	-0.07773093
iteration498	0.02218149	-0.3416391	-0.07755367
iteration499	0.02348518	-0.3361348	-0.07796609
iteration500	0.02491027	-0.3363833	-0.0777511
