# Supplementary material for: CoDaLoMic: An R package for modeling microbiome compositional and longitudinal data
Source: PLoS Comput Biol. 2026 Jun 22;22(6):e1014328. doi: 10.1371/journal.pcbi.1014328 (PMC13362355; doi:10.1371/journal.pcbi.1014328)
Supplement: S2 Table — FBM. Estimation quality. Value of the parameters in the last iterations of the optimization procedure to obtain the maximum likelihood estimation. The names of the parameters follow the notation in Equation 4. We can see that the values are identical, indicating that the optimization procedure has converged. (PDF) [file pcbi.1014328.s002.pdf]

Table S2. Simulated dataset. FBM. Estimation quality. Value of the parameters in the last iterations of the optimization procedure to obtain the maximum likelihood estimation. The names of the parameters follow the notation in Equation 4. We can see that the values are identical, indicating that the optimization procedure has converged.

Parameters	a_{11}	a_{12}	a_{13}	a_{21}	a_{22}	a_{23}
iteration195	0.4431963	-0.549043	0.352424	0.06766421	-0.2331457	0.07019841
iteration196	0.4431963	-0.549043	0.352424	0.06766421	-0.2331457	0.07019841
iteration197	0.4431963	-0.549043	0.352424	0.06766421	-0.2331457	0.07019841
iteration198	0.4431963	-0.549043	0.352424	0.06766421	-0.2331457	0.07019841
iteration199	0.4431963	-0.549043	0.352424	0.06766421	-0.2331457	0.07019841
iteration200	0.4431963	-0.549043	0.352424	0.06766421	-0.2331457	0.07019841
Parameters	a_{31}	a_{32}	a_{33}	a_{41}	a_{42}	a_{43}
iteration195	0.4715997	-0.8940771	0.3543566	-0.06588428	0.3402623	-0.7026974
iteration196	0.4715997	-0.8940771	0.3543566	-0.06588428	0.3402623	-0.7026974
iteration197	0.4715997	-0.8940771	0.3543566	-0.06588428	0.3402623	-0.7026974
iteration198	0.4715997	-0.8940771	0.3543566	-0.06588428	0.3402623	-0.7026974
iteration199	0.4715997	-0.8940771	0.3543566	-0.06588428	0.3402623	-0.7026974
iteration200	0.4715997	-0.8940771	0.3543566	-0.06588428	0.3402623	-0.7026974
Parameters	τ					
iteration195	12.18559					
iteration196	12.18559					
iteration197	12.18559					
iteration198	12.18559					
iteration199	12.18559					
iteration200	12.18559					
