# Supplementary material for: CoDaLoMic: An R package for modeling microbiome compositional and longitudinal data
Source: PLoS Comput Biol. 2026 Jun 22;22(6):e1014328. doi: 10.1371/journal.pcbi.1014328 (PMC13362355; doi:10.1371/journal.pcbi.1014328)
Supplement: S3 Table — BPBM. Estimation quality. Parameter information after obtaining the parameters of the BPBM model using MCMC. The first two columns represent the parameter names as described in Equation 5 and the corresponding names of the parameters outputted by R, respectively. The parameters that have a mean of zero, but non-zero values for the standard deviation and quantiles, are those whose credible intervals include zero at the center. The StudyingParam function has set their mean to zero. Since the estimated Rhat is less than 1.1 and the effective sample size (n.eff) exceeds 100, the quality of the estimation can be considered satisfactory. (PDF) [file pcbi.1014328.s003.pdf]

Table S3. Simulated dataset. BPBM. Estimation quality. Parameter information after obtaining the parameters of the BPBM model using MCMC. The first two columns represent the parameter names as described in Equation 5 and the corresponding names of the parameters outputted by R, respectively. The parameters that have a mean of zero, but non-zero values for the standard deviation and quantiles, are those whose credible intervals include zero at the center. The `StudyingParam` function has set their mean to zero. Since the estimated Rhat is less than 1.1 and the effective sample size (n.eff) exceeds 100, the quality of the estimation can be considered satisfactory.

name	R name	mean	sd	2.5%	25%	50%	75%	97.5%	Rhat	n.eff
a_{10}	a[1,1]	0.70	0.48	-0.18	0.36	0.72	1.03	1.61	1.00	8200.00
a_{20}	a[2,1]	0.00	0.44	-0.50	0.02	0.30	0.62	1.21	1.00	4200.00
a_{30}	a[3,1]	0.74	0.48	-0.17	0.41	0.75	1.07	1.64	1.00	2300.00
a_{40}	a[4,1]	0.00	0.39	-0.74	-0.13	0.07	0.34	0.86	1.00	5800.00
a_{50}	a[5,1]	0.64	0.47	-0.24	0.30	0.64	0.97	1.52	1.00	3600.00
a_{11}	a[1,2]	0.00	0.29	-0.65	-0.20	-0.02	0.11	0.54	1.00	3100.00
a_{21}	a[2,2]	0.28	0.29	-0.26	0.09	0.27	0.46	0.89	1.00	2300.00
a_{31}	a[3,2]	0.00	0.30	-0.50	-0.05	0.09	0.28	0.75	1.00	2100.00
a_{41}	a[4,2]	0.00	0.32	-0.82	-0.23	-0.04	0.10	0.52	1.00	2400.00
a_{51}	a[5,2]	0.00	0.27	-0.39	-0.02	0.13	0.30	0.72	1.00	2100.00
a_{12}	a[1,3]	0.00	0.41	-0.91	-0.21	0.00	0.20	0.80	1.00	1400.00
a_{22}	a[2,3]	0.00	0.42	-0.50	0.02	0.28	0.56	1.15	1.00	1800.00
a_{32}	a[3,3]	0.00	0.42	-0.88	-0.17	0.02	0.25	0.85	1.00	7200.00
a_{42}	a[4,3]	0.00	0.39	-0.77	-0.14	0.04	0.27	0.84	1.00	12000.00
a_{52}	a[5,3]	0.00	0.38	-0.60	-0.05	0.13	0.38	0.94	1.00	7000.00
a_{13}	a[1,4]	0.52	0.62	-0.49	0.07	0.44	0.90	1.90	1.01	610.00
a_{23}	a[2,4]	0.78	0.60	-0.21	0.35	0.74	1.16	2.10	1.01	570.00
a_{33}	a[3,4]	0.00	0.62	-1.15	-0.29	0.01	0.37	1.44	1.01	700.00
a_{43}	a[4,4]	0.89	0.75	-0.44	0.36	0.85	1.37	2.53	1.00	830.00
a_{53}	a[5,4]	0.00	0.50	-1.47	-0.75	-0.40	-0.08	0.53	1.00	930.00
	deviance	-46.98	9.62	-67.07	-53.10	-46.61	-40.42	-29.18	1.00	1100.00
σ_{10}	sdgamma[1,1]	1.99	1.33	0.13	0.88	1.72	2.97	4.76	1.00	12000.00
σ_{20}	sdgamma[2,1]	1.66	1.35	0.05	0.53	1.25	2.58	4.68	1.00	6000.00
σ_{30}	sdgamma[3,1]	2.02	1.33	0.14	0.92	1.77	3.00	4.73	1.00	10000.00
σ_{40}	sdgamma[4,1]	1.53	1.33	0.04	0.43	1.11	2.38	4.65	1.00	7100.00
σ_{50}	sdgamma[5,1]	1.94	1.34	0.10	0.82	1.64	2.91	4.75	1.00	3400.00
σ_{11}	sdgamma[1,2]	1.37	1.34	0.02	0.30	0.85	2.14	4.61	1.00	3500.00
σ_{21}	sdgamma[2,2]	1.57	1.34	0.05	0.47	1.14	2.42	4.64	1.00	12000.00
σ_{31}	sdgamma[3,2]	1.44	1.34	0.03	0.35	0.97	2.23	4.60	1.00	6800.00
σ_{41}	sdgamma[4,2]	1.41	1.33	0.03	0.33	0.91	2.18	4.57	1.00	3500.00
σ_{51}	sdgamma[5,2]	1.41	1.31	0.03	0.34	0.95	2.18	4.57	1.00	12000.00
σ_{12}	sdgamma[1,3]	1.46	1.33	0.03	0.38	1.02	2.26	4.62	1.00	12000.00
σ_{22}	sdgamma[2,3]	1.63	1.34	0.05	0.51	1.25	2.52	4.65	1.00	2800.00
σ_{32}	sdgamma[3,3]	1.46	1.33	0.03	0.36	1.02	2.28	4.61	1.00	4600.00
σ_{42}	sdgamma[4,3]	1.47	1.33	0.03	0.39	1.02	2.30	4.61	1.00	12000.00
σ_{52}	sdgamma[5,3]	1.50	1.33	0.04	0.41	1.07	2.35	4.61	1.00	5500.00
σ_{13}	sdgamma[1,4]	1.81	1.34	0.07	0.67	1.51	2.75	4.70	1.00	1100.00
σ_{23}	sdgamma[2,4]	2.02	1.34	0.13	0.90	1.77	3.04	4.77	1.00	1900.00
σ_{33}	sdgamma[3,4]	1.66	1.37	0.05	0.51	1.25	2.58	4.70	1.00	12000.00
σ_{43}	sdgamma[4,4]	2.11	1.36	0.13	0.96	1.88	3.17	4.79	1.00	3600.00
σ_{53}	sdgamma[5,4]	1.75	1.34	0.06	0.63	1.41	2.67	4.72	1.00	4500.00
