# Supplementary material for: CoDaLoMic: An R package for modeling microbiome compositional and longitudinal data
Source: PLoS Comput Biol. 2026 Jun 22;22(6):e1014328. doi: 10.1371/journal.pcbi.1014328 (PMC13362355; doi:10.1371/journal.pcbi.1014328)
Supplement: S4 Table — Dirich-gLV. Estimation quality. Parameter values from the final iterations of the optimization procedure to obtain the maximum likelihood estimation. Due to the high quantity of parameters, the information for all the parameters is in two tables, S4 and S5 Tables. We can see that the values are identical, indicating that the optimization procedure has converged. (PDF) [file pcbi.1014328.s004.pdf]

**Table S4.** Cockroach Dataset. Dirich-gLV. Estimation quality. Parameter values from the final iterations of the optimization procedure to obtain the maximum likelihood estimation. Due to the high quantity of parameters, the information for all the parameters is in two tables, Table S4 and Table S5. We can see that the values are identical, indicating that the optimization procedure has converged.

| interaction | $r_1$ | $r_2$ | $r_3$ | $r_4$ | $r_5$ | $r_6$ | $r_7$ | $r_8$ | $r_9$ | $r_{10}$ | $r_{11}$ | $r_{12}$ | $r_{13}$ | $r_{14}$ |
|---|---|---|---|---|---|---|---|---|---|---|---|---|---|---|
| iteration495 | 0.08345 | 11.60366 | 0.08987 | 11.92607 | -18.73651 | 1.86588 | 0.31173 | 1.86927 | -0.0969 | 1.54046 | 0.13831 | 2.22758 | -0.03017 | 2.22758 |
| iteration496 | 0.08345 | 11.60366 | 0.08987 | 11.92607 | -18.73651 | 1.86588 | 0.31173 | 1.86927 | -0.0969 | 1.54046 | 0.13831 | 2.22758 | -0.03017 | 2.22758 |
| iteration497 | 0.08345 | 11.60366 | 0.08987 | 11.92607 | -18.73651 | 1.86588 | 0.31173 | 1.86927 | -0.0969 | 1.54046 | 0.13831 | 2.22758 | -0.03017 | 2.22758 |
| iteration498 | 0.08345 | 11.60366 | 0.08987 | 11.92607 | -18.73651 | 1.86588 | 0.31173 | 1.86927 | -0.0969 | 1.54046 | 0.13831 | 2.22758 | -0.03017 | 2.22758 |
| iteration499 | 0.08345 | 11.60366 | 0.08987 | 11.92607 | -18.73651 | 1.86588 | 0.31173 | 1.86927 | -0.0969 | 1.54046 | 0.13831 | 2.22758 | -0.03017 | 2.22758 |
| iteration500 | 0.08345 | 11.60366 | 0.08987 | 11.92607 | -18.73651 | 1.86588 | 0.31173 | 1.86927 | -0.0969 | 1.54046 | 0.13831 | 2.22758 | -0.03017 | 2.22758 |
| interaction | $a_{1,1}$ | $a_{2,1}$ | $a_{3,1}$ | $a_{4,1}$ | $a_{5,1}$ | $a_{6,1}$ | $a_{7,1}$ | $a_{8,1}$ | $a_{9,1}$ | $a_{10,1}$ | $a_{11,1}$ | $a_{12,1}$ | $a_{13,1}$ | $a_{14,1}$ |
| iteration495 | 0.6588 | 11.97768 | 1.32884 | 11.94833 | 1.41647 | 11.95743 | 0.67134 | 1.53611 | 0.45902 | 1.88254 | 10.47341 | 1.45751 | 0.65547 | 1.21839 |
| iteration496 | 0.6588 | 11.97768 | 1.32884 | 11.94833 | 1.41647 | 11.95743 | 0.67134 | 1.53611 | 0.45902 | 1.88254 | 10.47341 | 1.45751 | 0.65547 | 1.21839 |
| iteration497 | 0.6588 | 11.97768 | 1.32884 | 11.94833 | 1.41647 | 11.95743 | 0.67134 | 1.53611 | 0.45902 | 1.88254 | 10.47341 | 1.45751 | 0.65547 | 1.21839 |
| iteration498 | 0.6588 | 11.97768 | 1.32884 | 11.94833 | 1.41647 | 11.95743 | 0.67134 | 1.53611 | 0.45902 | 1.88254 | 10.47341 | 1.45751 | 0.65547 | 1.21839 |
| iteration499 | 0.6588 | 11.97768 | 1.32884 | 11.94833 | 1.41647 | 11.95743 | 0.67134 | 1.53611 | 0.45902 | 1.88254 | 10.47341 | 1.45751 | 0.65547 | 1.21839 |
| iteration500 | 0.6588 | 11.97768 | 1.32884 | 11.94833 | 1.41647 | 11.95743 | 0.67134 | 1.53611 | 0.45902 | 1.88254 | 10.47341 | 1.45751 | 0.65547 | 1.21839 |
| interaction | $a_{1,2}$ | $a_{2,2}$ | $a_{3,2}$ | $a_{4,2}$ | $a_{5,2}$ | $a_{6,2}$ | $a_{7,2}$ | $a_{8,2}$ | $a_{9,2}$ | $a_{10,2}$ | $a_{11,2}$ | $a_{12,2}$ | $a_{13,2}$ | $a_{14,2}$ |
| iteration495 | 0.91774 | 0.9547 | 0.9018 | 2.22758 | 1.17945 | 12.27438 | 1.26734 | 0.91197 | -0.10682 | 0.95086 | 0.56822 | 0.6627 | 0.73097 | 0.44706 |
| iteration496 | 0.91774 | 0.9547 | 0.9018 | 2.22758 | 1.17945 | 12.27438 | 1.26734 | 0.91197 | -0.10682 | 0.95086 | 0.56822 | 0.6627 | 0.73097 | 0.44706 |
| iteration497 | 0.91774 | 0.9547 | 0.9018 | 2.22758 | 1.17945 | 12.27438 | 1.26734 | 0.91197 | -0.10682 | 0.95086 | 0.56822 | 0.6627 | 0.73097 | 0.44706 |
| iteration498 | 0.91774 | 0.9547 | 0.9018 | 2.22758 | 1.17945 | 12.27438 | 1.26734 | 0.91197 | -0.10682 | 0.95086 | 0.56822 | 0.6627 | 0.73097 | 0.44706 |
| iteration499 | 0.91774 | 0.9547 | 0.9018 | 2.22758 | 1.17945 | 12.27438 | 1.26734 | 0.91197 | -0.10682 | 0.95086 | 0.56822 | 0.6627 | 0.73097 | 0.44706 |
| iteration500 | 0.91774 | 0.9547 | 0.9018 | 2.22758 | 1.17945 | 12.27438 | 1.26734 | 0.91197 | -0.10682 | 0.95086 | 0.56822 | 0.6627 | 0.73097 | 0.44706 |
| interaction | $a_{1,3}$ | $a_{2,3}$ | $a_{3,3}$ | $a_{4,3}$ | $a_{5,3}$ | $a_{6,3}$ | $a_{7,3}$ | $a_{8,3}$ | $a_{9,3}$ | $a_{10,3}$ | $a_{11,3}$ | $a_{12,3}$ | $a_{13,3}$ | $a_{14,3}$ |
| iteration495 | 1.12115 | 0.97647 | 0.5155 | 2.22758 | 0.15653 | 1.93313 | 0.13291 | 0.7573 | 0.03939 | 0.80349 | 0.519 | 0.61334 | -0.53743 | 0.54788 |
| iteration496 | 1.12115 | 0.97647 | 0.5155 | 2.22758 | 0.15653 | 1.93313 | 0.13291 | 0.7573 | 0.03939 | 0.80349 | 0.519 | 0.61334 | -0.53743 | 0.54788 |
| iteration497 | 1.12115 | 0.97647 | 0.5155 | 2.22758 | 0.15653 | 1.93313 | 0.13291 | 0.7573 | 0.03939 | 0.80349 | 0.519 | 0.61334 | -0.53743 | 0.54788 |
| iteration498 | 1.12115 | 0.97647 | 0.5155 | 2.22758 | 0.15653 | 1.93313 | 0.13291 | 0.7573 | 0.03939 | 0.80349 | 0.519 | 0.61334 | -0.53743 | 0.54788 |
| iteration499 | 1.12115 | 0.97647 | 0.5155 | 2.22758 | 0.15653 | 1.93313 | 0.13291 | 0.7573 | 0.03939 | 0.80349 | 0.519 | 0.61334 | -0.53743 | 0.54788 |
| iteration500 | 1.12115 | 0.97647 | 0.5155 | 2.22758 | 0.15653 | 1.93313 | 0.13291 | 0.7573 | 0.03939 | 0.80349 | 0.519 | 0.61334 | -0.53743 | 0.54788 |
| interaction | $a_{1,4}$ | $a_{2,4}$ | $a_{3,4}$ | $a_{4,4}$ | $a_{5,4}$ | $a_{6,4}$ | $a_{7,4}$ | $a_{8,4}$ | $a_{9,4}$ | $a_{10,4}$ | $a_{11,4}$ | $a_{12,4}$ | $a_{13,4}$ | $a_{14,4}$ |
| iteration495 | 10.35302 | 1.93525 | 1.3949 | 2.22758 | 0.71746 | 2.22758 | 1.42836 | 1.53725 | 10.31023 | 1.64053 | 1.16082 | 0.74553 | 11.15295 | 0.49438 |
| iteration496 | 10.35302 | 1.93525 | 1.3949 | 2.22758 | 0.71746 | 2.22758 | 1.42836 | 1.53725 | 10.31023 | 1.64053 | 1.16082 | 0.74553 | 11.15295 | 0.49438 |
| iteration497 | 10.35302 | 1.93525 | 1.3949 | 2.22758 | 0.71746 | 2.22758 | 1.42836 | 1.53725 | 10.31023 | 1.64053 | 1.16082 | 0.74553 | 11.15295 | 0.49438 |
| iteration498 | 10.35302 | 1.93525 | 1.3949 | 2.22758 | 0.71746 | 2.22758 | 1.42836 | 1.53725 | 10.31023 | 1.64053 | 1.16082 | 0.74553 | 11.15295 | 0.49438 |
| iteration499 | 10.35302 | 1.93525 | 1.3949 | 2.22758 | 0.71746 | 2.22758 | 1.42836 | 1.53725 | 10.31023 | 1.64053 | 1.16082 | 0.74553 | 11.15295 | 0.49438 |
| iteration500 | 10.35302 | 1.93525 | 1.3949 | 2.22758 | 0.71746 | 2.22758 | 1.42836 | 1.53725 | 10.31023 | 1.64053 | 1.16082 | 0.74553 | 11.15295 | 0.49438 |
| interaction | $a_{1,5}$ | $a_{2,5}$ | $a_{3,5}$ | $a_{4,5}$ | $a_{5,5}$ | $a_{6,5}$ | $a_{7,5}$ | $a_{8,5}$ | $a_{9,5}$ | $a_{10,5}$ | $a_{11,5}$ | $a_{12,5}$ | $a_{13,5}$ | $a_{14,5}$ |
| iteration495 | 0.14334 | 1.12408 | 2.14648 | 2.22758 | 1.58401 | 2.22758 | 1.36904 | 0.90185 | 1.03264 | 0.95432 | 0.98969 | 0.49518 | 1.78474 | 0.37913 |
| iteration496 | 0.14334 | 1.12408 | 2.14648 | 2.22758 | 1.58401 | 2.22758 | 1.36904 | 0.90185 | 1.03264 | 0.95432 | 0.98969 | 0.49518 | 1.78474 | 0.37913 |
| iteration497 | 0.14334 | 1.12408 | 2.14648 | 2.22758 | 1.58401 | 2.22758 | 1.36904 | 0.90185 | 1.03264 | 0.95432 | 0.98969 | 0.49518 | 1.78474 | 0.37913 |
| iteration498 | 0.14334 | 1.12408 | 2.14648 | 2.22758 | 1.58401 | 2.22758 | 1.36904 | 0.90185 | 1.03264 | 0.95432 | 0.98969 | 0.49518 | 1.78474 | 0.37913 |
| iteration499 | 0.14334 | 1.12408 | 2.14648 | 2.22758 | 1.58401 | 2.22758 | 1.36904 | 0.90185 | 1.03264 | 0.95432 | 0.98969 | 0.49518 | 1.78474 | 0.37913 |
| iteration500 | 0.14334 | 1.12408 | 2.14648 | 2.22758 | 1.58401 | 2.22758 | 1.36904 | 0.90185 | 1.03264 | 0.95432 | 0.98969 | 0.49518 | 1.78474 | 0.37913 |
| interaction | $a_{1,6}$ | $a_{2,6}$ | $a_{3,6}$ | $a_{4,6}$ | $a_{5,6}$ | $a_{6,6}$ | $a_{7,6}$ | $a_{8,6}$ | $a_{9,6}$ | $a_{10,6}$ | $a_{11,6}$ | $a_{12,6}$ | $a_{13,6}$ | $a_{14,6}$ |
| iteration495 | 0.78824 | 1.91895 | 1.95452 | 2.22758 | -3.81813 | -17.90257 | 1.88215 | 1.15836 | 1.4399 | 1.16228 | 0.78042 | 0.51357 | 0.3468 | 0.51227 |
| iteration496 | 0.78824 | 1.91895 | 1.95452 | 2.22758 | -3.81813 | -17.90257 | 1.88215 | 1.15836 | 1.4399 | 1.16228 | 0.78042 | 0.51357 | 0.3468 | 0.51227 |
| iteration497 | 0.78824 | 1.91895 | 1.95452 | 2.22758 | -3.81813 | -17.90257 | 1.88215 | 1.15836 | 1.4399 | 1.16228 | 0.78042 | 0.51357 | 0.3468 | 0.51227 |
| iteration498 | 0.78824 | 1.91895 | 1.95452 | 2.22758 | -3.81813 | -17.90257 | 1.88215 | 1.15836 | 1.4399 | 1.16228 | 0.78042 | 0.51357 | 0.3468 | 0.51227 |
| iteration499 | 0.78824 | 1.91895 | 1.95452 | 2.22758 | -3.81813 | -17.90257 | 1.88215 | 1.15836 | 1.4399 | 1.16228 | 0.78042 | 0.51357 | 0.3468 | 0.51227 |
| iteration500 | 0.78824 | 1.91895 | 1.95452 | 2.22758 | -3.81813 | -17.90257 | 1.88215 | 1.15836 | 1.4399 | 1.16228 | 0.78042 | 0.51357 | 0.3468 | 0.51227 |
| interaction | $a_{1,7}$ | $a_{2,7}$ | $a_{3,7}$ | $a_{4,7}$ | $a_{5,7}$ | $a_{6,7}$ | $a_{7,7}$ | $a_{8,7}$ | $a_{9,7}$ | $a_{10,7}$ | $a_{11,7}$ | $a_{12,7}$ | $a_{13,7}$ | $a_{14,7}$ |
| iteration495 | 0.41736 | 1.14818 | 1.75198 | 2.22758 | 1.05624 | 2.22758 | 1.37084 | 0.90633 | 1.36081 | 0.9864 | 0.75786 | 0.55936 | 0.9233 | 0.5543 |
| iteration496 | 0.41736 | 1.14818 | 1.75198 | 2.22758 | 1.05624 | 2.22758 | 1.37084 | 0.90633 | 1.36081 | 0.9864 | 0.75786 | 0.55936 | 0.9233 | 0.5543 |
| iteration497 | 0.41736 | 1.14818 | 1.75198 | 2.22758 | 1.05624 | 2.22758 | 1.37084 | 0.90633 | 1.36081 | 0.9864 | 0.75786 | 0.55936 | 0.9233 | 0.5543 |
| iteration498 | 0.41736 | 1.14818 | 1.75198 | 2.22758 | 1.05624 | 2.22758 | 1.37084 | 0.90633 | 1.36081 | 0.9864 | 0.75786 | 0.55936 | 0.9233 | 0.5543 |
| iteration499 | 0.41736 | 1.14818 | 1.75198 | 2.22758 | 1.05624 | 2.22758 | 1.37084 | 0.90633 | 1.36081 | 0.9864 | 0.75786 | 0.55936 | 0.9233 | 0.5543 |
| iteration500 | 0.41736 | 1.14818 | 1.75198 | 2.22758 | 1.05624 | 2.22758 | 1.37084 | 0.90633 | 1.36081 | 0.9864 | 0.75786 | 0.55936 | 0.9233 | 0.5543 |
| interaction | $a_{1,8}$ | $a_{2,8}$ | $a_{3,8}$ | $a_{4,8}$ | $a_{5,8}$ | $a_{6,8}$ | $a_{7,8}$ | $a_{8,8}$ | $a_{9,8}$ | $a_{10,8}$ | $a_{11,8}$ | $a_{12,8}$ | $a_{13,8}$ | $a_{14,8}$ |
| iteration495 | 1.4084 | 0.99502 | 1.63004 | 2.22758 | 0.43718 | 1.93034 | 0.50684 | 0.68171 | -0.18372 | 0.81458 | 1.29447 | 0.49636 | -0.13896 | 0.57793 |
| iteration496 | 1.4084 | 0.99502 | 1.63004 | 2.22758 | 0.43718 | 1.93034 | 0.50684 | 0.68171 | -0.18372 | 0.81458 | 1.29447 | 0.49636 | -0.13896 | 0.57793 |
| iteration497 | 1.4084 | 0.99502 | 1.63004 | 2.22758 | 0.43718 | 1.93034 | 0.50684 | 0.68171 | -0.18372 | 0.81458 | 1.29447 | 0.49636 | -0.13896 | 0.57793 |
| iteration498 | 1.4084 | 0.99502 | 1.63004 | 2.22758 | 0.43718 | 1.93034 | 0.50684 | 0.68171 | -0.18372 | 0.81458 | 1.29447 | 0.49636 | -0.13896 | 0.57793 |
| iteration499 | 1.4084 | 0.99502 | 1.63004 | 2.22758 | 0.43718 | 1.93034 | 0.50684 | 0.68171 | -0.18372 | 0.81458 | 1.29447 | 0.49636 | -0.13896 | 0.57793 |
| iteration500 | 1.4084 | 0.99502 | 1.63004 | 2.22758 | 0.43718 | 1.93034 | 0.50684 | 0.68171 | -0.18372 | 0.81458 | 1.29447 | 0.49636 | -0.13896 | 0.57793 |
