# Supplementary material for: CoDaLoMic: An R package for modeling microbiome compositional and longitudinal data
Source: PLoS Comput Biol. 2026 Jun 22;22(6):e1014328. doi: 10.1371/journal.pcbi.1014328 (PMC13362355; doi:10.1371/journal.pcbi.1014328)
Supplement: S5 Table — Dirich-gLV. Estimation quality. Parameter values from the final iterations of the optimization procedure to obtain the maximum likelihood estimation. Due to the high quantity of parameters, the information for all the parameters is in two tables, S4 and S5 Tables. We can see that the values are identical, indicating that the optimization procedure has converged. (PDF) [file pcbi.1014328.s005.pdf]

**Table S5.** Cockroach Dataset. Dirich-gLV. Estimation quality. Parameter values from the final iterations of the optimization procedure to obtain the maximum likelihood estimation. Due to the high quantity of parameters, the information for all the parameters is in two tables, Table S4 and Table S5. We can see that the values are identical, indicating that the optimization procedure has converged.

| interaction | $a_{1,9}$ | $a_{2,9}$ | $a_{3,9}$ | $a_{4,9}$ | $a_{5,9}$ | $a_{6,9}$ | $a_{7,9}$ | $a_{8,9}$ | $a_{9,9}$ | $a_{10,9}$ | $a_{11,9}$ | $a_{12,9}$ | $a_{13,9}$ | $a_{14,9}$ |
|---|---|---|---|---|---|---|---|---|---|---|---|---|---|---|
| iteration495 | 21.05932 | 0.6822 | -0.54869 | 2.22758 | 0.39042 | 1.55348 | 0.79145 | 0.68386 | -0.97535 | 0.70762 | -0.5118 | 0.48221 | -0.55212 | 0.44472 |
| iteration496 | 21.05932 | 0.6822 | -0.54869 | 2.22758 | 0.39042 | 1.55348 | 0.79145 | 0.68386 | -0.97535 | 0.70762 | -0.5118 | 0.48221 | -0.55212 | 0.44472 |
| iteration497 | 21.05932 | 0.6822 | -0.54869 | 2.22758 | 0.39042 | 1.55348 | 0.79145 | 0.68386 | -0.97535 | 0.70762 | -0.5118 | 0.48221 | -0.55212 | 0.44472 |
| iteration498 | 21.05932 | 0.6822 | -0.54869 | 2.22758 | 0.39042 | 1.55348 | 0.79145 | 0.68386 | -0.97535 | 0.70762 | -0.5118 | 0.48221 | -0.55212 | 0.44472 |
| iteration499 | 21.05932 | 0.6822 | -0.54869 | 2.22758 | 0.39042 | 1.55348 | 0.79145 | 0.68386 | -0.97535 | 0.70762 | -0.5118 | 0.48221 | -0.55212 | 0.44472 |
| iteration500 | 21.05932 | 0.6822 | -0.54869 | 2.22758 | 0.39042 | 1.55348 | 0.79145 | 0.68386 | -0.97535 | 0.70762 | -0.5118 | 0.48221 | -0.55212 | 0.44472 |

| interaction | $a_{1,10}$ | $a_{2,10}$ | $a_{3,10}$ | $a_{4,10}$ | $a_{5,10}$ | $a_{6,10}$ | $a_{7,10}$ | $a_{8,10}$ | $a_{9,10}$ | $a_{10,10}$ | $a_{11,10}$ | $a_{12,10}$ | $a_{13,10}$ | $a_{14,10}$ |
|---|---|---|---|---|---|---|---|---|---|---|---|---|---|---|
| iteration495 | -0.02266 | 0.99983 | 1.11893 | 2.22758 | 1.62066 | 1.91964 | 1.20902 | 0.81679 | 1.02539 | 0.73277 | 1.04136 | 0.62134 | 0.69061 | 0.52167 |
| iteration496 | -0.02266 | 0.99983 | 1.11893 | 2.22758 | 1.62066 | 1.91964 | 1.20902 | 0.81679 | 1.02539 | 0.73277 | 1.04136 | 0.62134 | 0.69061 | 0.52167 |
| iteration497 | -0.02266 | 0.99983 | 1.11893 | 2.22758 | 1.62066 | 1.91964 | 1.20902 | 0.81679 | 1.02539 | 0.73277 | 1.04136 | 0.62134 | 0.69061 | 0.52167 |
| iteration498 | -0.02266 | 0.99983 | 1.11893 | 2.22758 | 1.62066 | 1.91964 | 1.20902 | 0.81679 | 1.02539 | 0.73277 | 1.04136 | 0.62134 | 0.69061 | 0.52167 |
| iteration499 | -0.02266 | 0.99983 | 1.11893 | 2.22758 | 1.62066 | 1.91964 | 1.20902 | 0.81679 | 1.02539 | 0.73277 | 1.04136 | 0.62134 | 0.69061 | 0.52167 |
| iteration500 | -0.02266 | 0.99983 | 1.11893 | 2.22758 | 1.62066 | 1.91964 | 1.20902 | 0.81679 | 1.02539 | 0.73277 | 1.04136 | 0.62134 | 0.69061 | 0.52167 |

| interaction | $a_{1,11}$ | $a_{2,11}$ | $a_{3,11}$ | $a_{4,11}$ | $a_{5,11}$ | $a_{6,11}$ | $a_{7,11}$ | $a_{8,11}$ | $a_{9,11}$ | $a_{10,11}$ | $a_{11,11}$ | $a_{12,11}$ | $a_{13,11}$ | $a_{14,11}$ |
|---|---|---|---|---|---|---|---|---|---|---|---|---|---|---|
| iteration495 | -18.04812 | 0.88043 | -0.14233 | 2.22758 | -0.30811 | 1.57423 | 0.88109 | 0.68898 | 0.15812 | 0.78084 | 1.22537 | 0.5687 | -0.19101 | 0.52093 |
| iteration496 | -18.04812 | 0.88043 | -0.14233 | 2.22758 | -0.30811 | 1.57423 | 0.88109 | 0.68898 | 0.15812 | 0.78084 | 1.22537 | 0.5687 | -0.19101 | 0.52093 |
| iteration497 | -18.04812 | 0.88043 | -0.14233 | 2.22758 | -0.30811 | 1.57423 | 0.88109 | 0.68898 | 0.15812 | 0.78084 | 1.22537 | 0.5687 | -0.19101 | 0.52093 |
| iteration498 | -18.04812 | 0.88043 | -0.14233 | 2.22758 | -0.30811 | 1.57423 | 0.88109 | 0.68898 | 0.15812 | 0.78084 | 1.22537 | 0.5687 | -0.19101 | 0.52093 |
| iteration499 | -18.04812 | 0.88043 | -0.14233 | 2.22758 | -0.30811 | 1.57423 | 0.88109 | 0.68898 | 0.15812 | 0.78084 | 1.22537 | 0.5687 | -0.19101 | 0.52093 |
| iteration500 | -18.04812 | 0.88043 | -0.14233 | 2.22758 | -0.30811 | 1.57423 | 0.88109 | 0.68898 | 0.15812 | 0.78084 | 1.22537 | 0.5687 | -0.19101 | 0.52093 |

| interaction | $a_{1,12}$ | $a_{2,12}$ | $a_{3,12}$ | $a_{4,12}$ | $a_{5,12}$ | $a_{6,12}$ | $a_{7,12}$ | $a_{8,12}$ | $a_{9,12}$ | $a_{10,12}$ | $a_{11,12}$ | $a_{12,12}$ | $a_{13,12}$ | $a_{14,12}$ |
|---|---|---|---|---|---|---|---|---|---|---|---|---|---|---|
| iteration495 | 1.20448 | 0.78316 | -0.05422 | 2.22758 | 1.60206 | 1.59162 | 1.28385 | 0.7143 | -0.71225 | 0.6757 | -0.65362 | 0.46012 | 0.0939 | 0.4627 |
| iteration496 | 1.20448 | 0.78316 | -0.05422 | 2.22758 | 1.60206 | 1.59162 | 1.28385 | 0.7143 | -0.71225 | 0.6757 | -0.65362 | 0.46012 | 0.0939 | 0.4627 |
| iteration497 | 1.20448 | 0.78316 | -0.05422 | 2.22758 | 1.60206 | 1.59162 | 1.28385 | 0.7143 | -0.71225 | 0.6757 | -0.65362 | 0.46012 | 0.0939 | 0.4627 |
| iteration498 | 1.20448 | 0.78316 | -0.05422 | 2.22758 | 1.60206 | 1.59162 | 1.28385 | 0.7143 | -0.71225 | 0.6757 | -0.65362 | 0.46012 | 0.0939 | 0.4627 |
| iteration499 | 1.20448 | 0.78316 | -0.05422 | 2.22758 | 1.60206 | 1.59162 | 1.28385 | 0.7143 | -0.71225 | 0.6757 | -0.65362 | 0.46012 | 0.0939 | 0.4627 |
| iteration500 | 1.20448 | 0.78316 | -0.05422 | 2.22758 | 1.60206 | 1.59162 | 1.28385 | 0.7143 | -0.71225 | 0.6757 | -0.65362 | 0.46012 | 0.0939 | 0.4627 |

| interaction | $a_{1,13}$ | $a_{2,13}$ | $a_{3,13}$ | $a_{4,13}$ | $a_{5,13}$ | $a_{6,13}$ | $a_{7,13}$ | $a_{8,13}$ | $a_{9,13}$ | $a_{10,13}$ | $a_{11,13}$ | $a_{12,13}$ | $a_{13,13}$ | $a_{14,13}$ |
|---|---|---|---|---|---|---|---|---|---|---|---|---|---|---|
| iteration495 | 0.95671 | 0.72178 | 0.39295 | 2.22758 | 0.68066 | 1.54435 | 1.54404 | 0.69909 | 0.55703 | 0.6747 | 0.60265 | 0.46441 | -0.56456 | 0.42943 |
| iteration496 | 0.95671 | 0.72178 | 0.39295 | 2.22758 | 0.68066 | 1.54435 | 1.54404 | 0.69909 | 0.55703 | 0.6747 | 0.60265 | 0.46441 | -0.56456 | 0.42943 |
| iteration497 | 0.95671 | 0.72178 | 0.39295 | 2.22758 | 0.68066 | 1.54435 | 1.54404 | 0.69909 | 0.55703 | 0.6747 | 0.60265 | 0.46441 | -0.56456 | 0.42943 |
| iteration498 | 0.95671 | 0.72178 | 0.39295 | 2.22758 | 0.68066 | 1.54435 | 1.54404 | 0.69909 | 0.55703 | 0.6747 | 0.60265 | 0.46441 | -0.56456 | 0.42943 |
| iteration499 | 0.95671 | 0.72178 | 0.39295 | 2.22758 | 0.68066 | 1.54435 | 1.54404 | 0.69909 | 0.55703 | 0.6747 | 0.60265 | 0.46441 | -0.56456 | 0.42943 |
| iteration500 | 0.95671 | 0.72178 | 0.39295 | 2.22758 | 0.68066 | 1.54435 | 1.54404 | 0.69909 | 0.55703 | 0.6747 | 0.60265 | 0.46441 | -0.56456 | 0.42943 |

| interaction | $a_{1,14}$ | $a_{2,14}$ | $a_{3,14}$ | $a_{4,14}$ | $a_{5,14}$ | $a_{6,14}$ | $a_{7,14}$ | $a_{8,14}$ | $a_{9,14}$ | $a_{10,14}$ | $a_{11,14}$ | $a_{12,14}$ | $a_{13,14}$ | $a_{14,14}$ |
|---|---|---|---|---|---|---|---|---|---|---|---|---|---|---|
| iteration495 | 1.09807 | 0.88702 | -0.22318 | 2.22758 | 0.28507 | 1.58405 | 0.92004 | 0.61195 | 0.01898 | 0.73848 | -0.31736 | 0.49619 | 0.20559 | 0.49005 |
| iteration496 | 1.09807 | 0.88702 | -0.22318 | 2.22758 | 0.28507 | 1.58405 | 0.92004 | 0.61195 | 0.01898 | 0.73848 | -0.31736 | 0.49619 | 0.20559 | 0.49005 |
| iteration497 | 1.09807 | 0.88702 | -0.22318 | 2.22758 | 0.28507 | 1.58405 | 0.92004 | 0.61195 | 0.01898 | 0.73848 | -0.31736 | 0.49619 | 0.20559 | 0.49005 |
| iteration498 | 1.09807 | 0.88702 | -0.22318 | 2.22758 | 0.28507 | 1.58405 | 0.92004 | 0.61195 | 0.01898 | 0.73848 | -0.31736 | 0.49619 | 0.20559 | 0.49005 |
| iteration499 | 1.09807 | 0.88702 | -0.22318 | 2.22758 | 0.28507 | 1.58405 | 0.92004 | 0.61195 | 0.01898 | 0.73848 | -0.31736 | 0.49619 | 0.20559 | 0.49005 |
| iteration500 | 1.09807 | 0.88702 | -0.22318 | 2.22758 | 0.28507 | 1.58405 | 0.92004 | 0.61195 | 0.01898 | 0.73848 | -0.31736 | 0.49619 | 0.20559 | 0.49005 |

| interaction | $\tau$ |
|---|---|
| interaction495 | 100.46796 |
| interaction496 | 100.46796 |
| interaction497 | 100.46796 |
| interaction498 | 100.46796 |
| interaction499 | 100.46796 |
| interaction500 | 100.46796 |
