# Supplementary material for: CoDaLoMic: An R package for modeling microbiome compositional and longitudinal data
Source: PLoS Comput Biol. 2026 Jun 22;22(6):e1014328. doi: 10.1371/journal.pcbi.1014328 (PMC13362355; doi:10.1371/journal.pcbi.1014328)
Supplement: S6 Table — FBM. Estimation quality. Parameter values from the final iterations of the optimization procedure to obtain the maximum likelihood estimation. The parameters are named according to the notation in Equation 4. Since the values are the same, it indicates that the optimization procedure has converged. (PDF) [file pcbi.1014328.s006.pdf]

Table S6. Cockroach dataset. FBM. Estimation quality. Parameter values from the final iterations of the optimization procedure to obtain the maximum likelihood estimation. The parameters are named according to the notation in Equation 4. Since the values are the same, it indicates that the optimization procedure has converged.

iteration	$a_{1,1}$	$a_{1,2}$	$a_{1,3}$	$a_{2,1}$	$a_{2,2}$	$a_{2,3}$	$a_{3,1}$	$a_{3,2}$	$a_{3,3}$	$a_{4,1}$	$a_{4,2}$
iteration295	0.9142406289	0.1210112612	0.4036853091	-0.3338606182	0.1634659976	0.4250194417	-0.2853568797	0.6589539581	0.1235090926	-0.0563446588	0.2610542856
iteration296	0.9142406289	0.1210112612	0.4036853091	-0.3338606182	0.1634659976	0.4250194417	-0.2853568797	0.6589539581	0.1235090926	-0.0563446588	0.2610542856
iteration297	0.9142406289	0.1210112612	0.4036853091	-0.3338606182	0.1634659976	0.4250194417	-0.2853568797	0.6589539581	0.1235090926	-0.0563446588	0.2610542856
iteration298	0.9142406289	0.1210112612	0.4036853091	-0.3338606182	0.1634659976	0.4250194417	-0.2853568797	0.6589539581	0.1235090926	-0.0563446588	0.2610542856
iteration299	0.9142406289	0.1210112612	0.4036853091	-0.3338606182	0.1634659976	0.4250194417	-0.2853568797	0.6589539581	0.1235090926	-0.0563446588	0.2610542856
iteration300	0.9142406289	0.1210112612	0.4036853091	-0.3338606182	0.1634659976	0.4250194417	-0.2853568797	0.6589539581	0.1235090926	-0.0563446588	0.2610542856

iteration	$a_{4,3}$	$a_{5,1}$	$a_{5,2}$	$a_{5,3}$	$a_{6,1}$	$a_{6,2}$	$a_{6,3}$	$a_{7,1}$	$a_{7,2}$	$a_{7,3}$	$a_{8,1}$
iteration295	0.1829964264	0.7683095403	0.5984186146	0.5220263907	-0.0169165657	0.362796398	0.2926015412	0.6921735491	0.346348288	0.6382797681	-0.3683730325
iteration296	0.1829964264	0.7683095403	0.5984186146	0.5220263907	-0.0169165657	0.362796398	0.2926015412	0.6921735491	0.346348288	0.6382797681	-0.3683730325
iteration297	0.1829964264	0.7683095403	0.5984186146	0.5220263907	-0.0169165657	0.362796398	0.2926015412	0.6921735491	0.346348288	0.6382797681	-0.3683730325
iteration298	0.1829964264	0.7683095403	0.5984186146	0.5220263907	-0.0169165657	0.362796398	0.2926015412	0.6921735491	0.346348288	0.6382797681	-0.3683730325
iteration299	0.1829964264	0.7683095403	0.5984186146	0.5220263907	-0.0169165657	0.362796398	0.2926015412	0.6921735491	0.346348288	0.6382797681	-0.3683730325
iteration300	0.1829964264	0.7683095403	0.5984186146	0.5220263907	-0.0169165657	0.362796398	0.2926015412	0.6921735491	0.346348288	0.6382797681	-0.3683730325

iteration	$a_{8,2}$	$a_{8,3}$	$a_{9,1}$	$a_{9,2}$	$a_{9,3}$	$a_{10,1}$	$a_{10,2}$	$a_{10,3}$	$a_{11,1}$	$a_{11,2}$	$a_{11,3}$
iteration295	0.4557605817	0.2646997504	-1.6318350651	0.1285295801	0.408961944	-0.0060784327	0.5205238371	0.3316187299	-1.6030359507	0.1805614177	0.092078744
iteration296	0.4557605817	0.2646997504	-1.6318350651	0.1285295801	0.408961944	-0.0060784327	0.5205238371	0.3316187299	-1.6030359507	0.1805614177	0.092078744
iteration297	0.4557605817	0.2646997504	-1.6318350651	0.1285295801	0.408961944	-0.0060784327	0.5205238371	0.3316187299	-1.6030359507	0.1805614177	0.092078744
iteration298	0.4557605817	0.2646997504	-1.6318350651	0.1285295801	0.408961944	-0.0060784327	0.5205238371	0.3316187299	-1.6030359507	0.1805614177	0.092078744
iteration299	0.4557605817	0.2646997504	-1.6318350651	0.1285295801	0.408961944	-0.0060784327	0.5205238371	0.3316187299	-1.6030359507	0.1805614177	0.092078744
iteration300	0.4557605817	0.2646997504	-1.6318350651	0.1285295801	0.408961944	-0.0060784327	0.5205238371	0.3316187299	-1.6030359507	0.1805614177	0.092078744

iteration	$a_{12,1}$	$a_{12,2}$	$a_{12,3}$	$a_{13,1}$	$a_{13,2}$	$a_{13,3}$	$a_{14,1}$	$a_{14,2}$	$a_{14,3}$	tau	
iteration295	-1.1354379015	0.1023258884	0.5145126572	-1.8314347618	0.105270793	0.3434478877	-1.0553661597	0.3422324828	0.1563316788	59.9786503461	
iteration296	-1.1354379015	0.1023258884	0.5145126572	-1.8314347618	0.105270793	0.3434478877	-1.0553661597	0.3422324828	0.1563316788	59.9786503461	
iteration297	-1.1354379015	0.1023258884	0.5145126572	-1.8314347618	0.105270793	0.3434478877	-1.0553661597	0.3422324828	0.1563316788	59.9786503461	
iteration298	-1.1354379015	0.1023258884	0.5145126572	-1.8314347618	0.105270793	0.3434478877	-1.0553661597	0.3422324828	0.1563316788	59.9786503461	
iteration299	-1.1354379015	0.1023258884	0.5145126572	-1.8314347618	0.105270793	0.3434478877	-1.0553661597	0.3422324828	0.1563316788	59.9786503461	
iteration300	-1.1354379015	0.1023258884	0.5145126572	-1.8314347618	0.105270793	0.3434478877	-1.0553661597	0.3422324828	0.1563316788	59.9786503461	
