# Supplementary material for: CoDaLoMic: An R package for modeling microbiome compositional and longitudinal data
Source: PLoS Comput Biol. 2026 Jun 22;22(6):e1014328. doi: 10.1371/journal.pcbi.1014328 (PMC13362355; doi:10.1371/journal.pcbi.1014328)
Supplement: S7 Table — BPBM. Estimation quality. Parameter information after obtaining the parameters of the BPBM model using MCMC. Due to the high quantity of parameters, the information for all the parameters is in two tables, S7 and S8 Tables. The parameters with a mean of zero, but non-zero values for the standard deviation and quantiles, are those whose credible intervals include zero at the center. The StudyingParam function has adjusted their mean to zero. Since the estimated Rhat is less than 1.1 and the effective sample size (n.eff) exceeds 100, the quality of the estimation can be considered satisfactory. (PDF) [file pcbi.1014328.s007.pdf]

Table S7. Cockroach dataset. BPBM. Estimation quality. Parameter information after obtaining the parameters of the BPBM model using MCMC. Due to the high quantity of parameters, the information for all the parameters is in two tables, Table S7 and Table S8. The parameters with a mean of zero, but non-zero values for the standard deviation and quantiles, are those whose credible intervals include zero at the center. The `StudyingParam` function has adjusted their mean to zero. Since the estimated Rhat is less than 1.1 and the effective sample size (n.eff) exceeds 100, the quality of the estimation can be considered satisfactory.

	mean	sd	2.5%	25%	50%	75%	97.5%	Rhat	n.eff		mean	sd	2.5%	25%	50%	75%	97.5%	Rhat	n.eff
a[1,1]	2.48	0.28	1.9	2.3	2.5	2.68	2.99	1.01	360	sdgamma[1,1]	3.04	1.08	1.17	2.15	3.01	3.94	4.89	1	27000
a[2,1]	1.36	0.44	0.5	1.06	1.36	1.66	2.22	1	1100	sdgamma[2,1]	2.49	1.23	0.58	1.46	2.35	3.45	4.83	1	11000
a[3,1]	0.65	0.42	-0.1	0.36	0.64	0.92	1.51	1.01	390	sdgamma[3,1]	1.94	1.34	0.13	0.82	1.64	2.92	4.75	1	2200
a[4,1]	1.82	0.32	1.15	1.61	1.83	2.04	2.38	1.01	410	sdgamma[4,1]	2.76	1.16	0.89	1.8	2.66	3.7	4.87	1	5700
a[5,1]	1.18	0.36	0.44	0.95	1.18	1.42	1.88	1	910	sdgamma[5,1]	2.39	1.24	0.53	1.34	2.21	3.35	4.81	1	7100
a[6,1]	1.28	0.34	0.58	1.06	1.3	1.51	1.9	1.01	340	sdgamma[6,1]	2.46	1.23	0.61	1.42	2.3	3.43	4.82	1	6500
a[7,1]	1.25	0.37	0.47	1.02	1.26	1.49	1.93	1.01	330	sdgamma[7,1]	2.43	1.24	0.56	1.38	2.28	3.4	4.81	1	1700
a[8,1]	0	0.36	-0.47	0	0.22	0.48	0.97	1	1800	sdgamma[8,1]	1.57	1.34	0.04	0.46	1.13	2.43	4.64	1	4900
a[9,1]	0	0.43	-1.02	-0.37	-0.08	0.13	0.76	1	1900	sdgamma[9,1]	1.54	1.34	0.04	0.44	1.11	2.41	4.67	1	9000
a[10,1]	0.47	0.41	-0.3	0.18	0.47	0.74	1.33	1	14000	sdgamma[10,1]	1.76	1.34	0.07	0.66	1.41	2.69	4.73	1	27000
a[11,1]	0	0.4	-1.06	-0.35	-0.07	0.1	0.59	1	640	sdgamma[11,1]	1.48	1.34	0.03	0.39	1.03	2.3	4.65	1	3500
a[12,1]	-0.38	0.5	-1.5	-0.68	-0.29	-0.02	0.43	1	1700	sdgamma[12,1]	1.69	1.37	0.05	0.54	1.32	2.63	4.7	1	9100
a[13,1]	0	0.45	-1.29	-0.58	-0.26	-0.01	0.5	1	19000	sdgamma[13,1]	1.64	1.35	0.05	0.51	1.24	2.54	4.67	1	27000
a[14,1]	0	0.42	-0.88	-0.27	-0.02	0.22	0.87	1	5100	sdgamma[14,1]	1.52	1.34	0.04	0.42	1.07	2.36	4.64	1	22000
a[15,1]	3.14	0.29	2.57	2.94	3.15	3.34	3.68	1.01	320	sdgamma[15,1]	3.26	1.01	1.42	2.45	3.29	4.12	4.9	1	25000
a[1,2]	-0.1	0.05	-0.2	-0.14	-0.1	-0.07	-0.01	1	5700	sdgamma[1,2]	1.2	1.26	0.04	0.23	0.67	1.78	4.48	1	11000
a[2,2]	0	0.07	-0.2	-0.1	-0.05	0	0.07	1	4300	sdgamma[2,2]	1.06	1.25	0.01	0.15	0.5	1.59	4.4	1	7800
a[3,2]	0	0.07	-0.11	-0.02	0.02	0.06	0.16	1	3300	sdgamma[3,2]	1.02	1.24	0.01	0.12	0.44	1.49	4.41	1	27000
a[4,2]	-0.05	0.05	-0.16	-0.08	-0.04	-0.01	0.05	1	8700	sdgamma[4,2]	1.03	1.25	0.01	0.13	0.46	1.53	4.43	1	27000
a[5,2]	-0.06	0.06	-0.2	-0.11	-0.06	-0.01	0.05	1	9100	sdgamma[5,2]	1.05	1.24	0.01	0.15	0.5	1.55	4.43	1	4100
a[6,2]	-0.07	0.06	-0.19	-0.11	-0.07	-0.03	0.03	1	1800	sdgamma[6,2]	1.09	1.26	0.02	0.16	0.53	1.62	4.49	1	12000
a[7,2]	0	0.06	-0.15	-0.06	-0.02	0.01	0.08	1	2200	sdgamma[7,2]	1.01	1.24	0.01	0.12	0.43	1.48	4.4	1	25000
a[8,2]	0	0.07	-0.19	-0.1	-0.05	0	0.07	1	5900	sdgamma[8,2]	1.07	1.26	0.01	0.14	0.49	1.59	4.44	1	27000
a[9,2]	0	0.09	-0.11	0	0.05	0.11	0.23	1	27000	sdgamma[9,2]	1.12	1.27	0.01	0.16	0.55	1.67	4.47	1	27000
a[10,2]	-0.12	0.08	-0.27	-0.17	-0.12	-0.06	0.02	1	17000	sdgamma[10,2]	1.24	1.29	0.03	0.24	0.7	1.88	4.52	1	19000
a[11,2]	-0.13	0.08	-0.29	-0.18	-0.12	-0.07	0.02	1	2200	sdgamma[11,2]	1.27	1.29	0.03	0.26	0.75	1.95	4.54	1	7200
a[12,2]	0	0.08	-0.17	-0.05	0	0.05	0.16	1	5900	sdgamma[12,2]	1.04	1.24	0.01	0.13	0.47	1.51	4.43	1	27000
a[13,2]	0	0.09	-0.23	-0.1	-0.03	0.01	0.13	1	27000	sdgamma[13,2]	1.1	1.28	0.01	0.15	0.53	1.65	4.48	1	27000
a[14,2]	0	0.08	-0.21	-0.09	-0.03	0.02	0.13	1	25000	sdgamma[14,2]	1.06	1.26	0.01	0.14	0.49	1.57	4.49	1	21000
a[15,2]	-0.08	0.05	-0.18	-0.11	-0.08	-0.04	0.01	1	2700	sdgamma[15,2]	1.12	1.26	0.02	0.18	0.56	1.68	4.49	1	27000
a[1,3]	-0.06	0.06	-0.19	-0.1	-0.06	-0.01	0.06	1	7000	sdgamma[1,3]	1.09	1.27	0.01	0.15	0.52	1.62	4.5	1	25000
a[2,3]	0	0.1	-0.15	-0.02	0.03	0.1	0.25	1	2800	sdgamma[2,3]	1.09	1.25	0.01	0.16	0.54	1.63	4.46	1	27000
a[3,3]	0	0.1	-0.12	0	0.06	0.14	0.29	1	2800	sdgamma[3,3]	1.17	1.29	0.02	0.19	0.6	1.76	4.5	1	9500
a[4,3]	-0.1	0.07	-0.24	-0.15	-0.1	-0.05	0.03	1	2500	sdgamma[4,3]	1.19	1.29	0.03	0.22	0.64	1.79	4.54	1	18000
a[5,3]	0	0.09	-0.24	-0.12	-0.06	0	0.1	1	9000	sdgamma[5,3]	1.13	1.28	0.01	0.17	0.56	1.71	4.49	1	16000
a[6,3]	0	0.08	-0.21	-0.1	-0.05	0	0.09	1	9100	sdgamma[6,3]	1.07	1.25	0.01	0.15	0.51	1.59	4.47	1	27000
a[7,3]	-0.12	0.09	-0.3	-0.19	-0.12	-0.06	0.04	1	4800	sdgamma[7,3]	1.26	1.3	0.03	0.25	0.73	1.93	4.55	1	6600
a[8,3]	0	0.09	-0.2	-0.07	-0.01	0.03	0.16	1	27000	sdgamma[8,3]	1.07	1.25	0.01	0.15	0.51	1.57	4.47	1	14000
a[9,3]	0	0.12	-0.24	-0.07	0	0.08	0.26	1	7200	sdgamma[9,3]	1.13	1.27	0.02	0.17	0.58	1.7	4.48	1	27000
a[10,3]	0	0.1	-0.23	-0.09	-0.02	0.04	0.18	1	5300	sdgamma[10,3]	1.09	1.25	0.01	0.16	0.54	1.64	4.46	1	9000
a[11,3]	0	0.11	-0.19	-0.04	0.02	0.1	0.26	1	9900	sdgamma[11,3]	1.13	1.26	0.01	0.17	0.57	1.7	4.45	1	19000
a[12,3]	0	0.11	-0.21	-0.06	0	0.08	0.24	1	11000	sdgamma[12,3]	1.12	1.27	0.01	0.17	0.56	1.68	4.48	1	27000
a[13,3]	0	0.13	-0.28	-0.1	-0.02	0.05	0.23	1	21000	sdgamma[13,3]	1.15	1.29	0.02	0.19	0.59	1.74	4.52	1	22000
a[14,3]	0	0.13	-0.3	-0.12	-0.03	0.03	0.21	1	27000	sdgamma[14,3]	1.16	1.29	0.02	0.19	0.6	1.76	4.51	1	5600
a[15,3]	0	0.06	-0.09	-0.01	0.03	0.07	0.15	1	2300	sdgamma[15,3]	1.01	1.24	0.01	0.12	0.44	1.49	4.44	1	8400
a[1,4]	0.18	0.07	0.03	0.13	0.18	0.23	0.32	1	8900	sdgamma[1,4]	1.38	1.3	0.08	0.35	0.88	2.11	4.58	1	27000
a[2,4]	0.15	0.12	-0.07	0.06	0.14	0.23	0.39	1	9400	sdgamma[2,4]	1.31	1.31	0.03	0.28	0.79	2.02	4.57	1	5900
a[3,4]	0.2	0.1	0	0.12	0.2	0.27	0.4	1	3300	sdgamma[3,4]	1.41	1.31	0.07	0.37	0.91	2.16	4.6	1	27000
a[4,4]	0	0.07	-0.17	-0.07	-0.02	0.02	0.12	1	4000	sdgamma[4,4]	1.02	1.24	0.01	0.13	0.45	1.49	4.42	1	23000
a[5,4]	0.16	0.1	-0.02	0.09	0.16	0.23	0.35	1	5300	sdgamma[5,4]	1.32	1.3	0.05	0.31	0.81	2.04	4.57	1	20000
a[6,4]	0.16	0.09	0	0.1	0.16	0.22	0.34	1	9000	sdgamma[6,4]	1.34	1.3	0.05	0.32	0.83	2.03	4.58	1	23000
a[7,4]	0	0.09	-0.18	-0.06	0	0.05	0.17	1	2600	sdgamma[7,4]	1.06	1.25	0.01	0.14	0.49	1.57	4.46	1	27000
a[8,4]	0.27	0.11	0.05	0.19	0.27	0.34	0.48	1	27000	sdgamma[8,4]	1.55	1.31	0.11	0.47	1.09	2.37	4.64	1	11000
a[9,4]	0	0.12	-0.2	-0.04	0.03	0.11	0.28	1	25000	sdgamma[9,4]	1.14	1.27	0.02	0.19	0.6	1.71	4.5	1	27000
a[10,4]	0.16	0.11	-0.04	0.08	0.16	0.24	0.38	1	4500	sdgamma[10,4]	1.33	1.3	0.04	0.31	0.82	2.02	4.57	1	27000
a[11,4]	0.22	0.13	-0.01	0.14	0.22	0.31	0.48	1	27000	sdgamma[11,4]	1.47	1.32	0.07	0.4	0.99	2.27	4.62	1	27000
a[12,4]	0	0.12	-0.18	-0.02	0.04	0.13	0.31	1	7300	sdgamma[12,4]	1.15	1.27	0.02	0.19	0.6	1.74	4.5	1	27000
a[13,4]	0.12	0.14	-0.13	0.02	0.11	0.21	0.4	1	17000	sdgamma[13,4]	1.28	1.3	0.02	0.26	0.76	1.98	4.54	1	14000
a[14,4]	0	0.13	-0.13	0.02	0.1	0.2	0.38	1	16000	sdgamma[14,4]	1.25	1.29	0.02	0.25	0.71	1.92	4.53	1	14000
a[15,4]	0.18	0.08	0.02	0.13	0.18	0.23	0.33	1	3600	sdgamma[15,4]	1.39	1.31	0.08	0.35	0.89	2.13	4.6	1	14000
a[1,5]	-0.13	0.06	-0.24	-0.17	-0.13	-0.09	-0.01	1	4300	sdgamma[1,5]	1.28	1.29	0.06	0.27	0.74	1.94	4.55	1	21000
a[2,5]	0	0.08	-0.12	-0.01	0.03	0.09	0.21	1	12000	sdgamma[2,5]	1.08	1.27	0.01	0.15	0.51	1.61	4.49	1	27000
a[3,5]	0	0.09	-0.25	-0.12	-0.05	0	0.11	1	16000	sdgamma[3,5]	1.12	1.28	0.01	0.16	0.55	1.69	4.5	1	27000
a[4,5]	0.06	0.07	-0.06	0.01	0.06	0.1	0.2	1	10000	sdgamma[4,5]	1.09	1.26	0.01	0.16	0.52	1.63	4.47	1	10000
a[5,5]	0	0.07	-0.14	-0.03	0.01	0.06	0.17	1	15000	sdgamma[5,5]	1.02	1.24	0.01	0.13	0.45	1.49	4.41	1	18000
a[6,5]	0	0.07	-0.13	-0.03	0.01	0.06	0.16	1	9600	sdgamma[6,5]	1.02	1.24	0.01	0.12	0.45	1.49	4.43	1	8400
a[7,5]	0.07	0.08	-0.07	0.02	0.07	0.12	0.23	1	7500	sdgamma[7,5]	1.13	1.27	0.01	0.18	0.57	1.72	4.49	1	14000
a[8,5]	0	0.08	-0.17	-0.05	0	0.05	0.17	1	27000	sdgamma[8,5]	1.05	1.25	0.01	0.14	0.49	1.56	4.46	1	9800
a[9,5]	0	0.11	-0.3	-0.14	-0.06	0.01	0.14	1	18000	sdgamma[9,5]	1.16	1.28	0.02	0.19	0.6	1.76	4.53	1	15000
a[10,5]	0	0.09	-0.16	-0.03	0.02	0.08	0.21	1	10000	sdgamma[10,5]	1.07	1.26	0.01	0.14	0.51	1.57	4.47	1	27000
a[11,5]	0	0.1	-0.13	-0.01	0.05	0.12	0.27	1	27000	sdgamma[11,5]	1.13	1.26	0.01	0.17	0.58	1.7	4.47	1	10000
a[12,5]	0	0.11	-0.28	-0.12	-0.04	0.02	0.16	1	25000	sdgamma[12,5]	1.15	1.28	0.02	0.18	0.59	1.73	4.51	1	17000
a[13,5]	0	0.11	-0.24	-0.07	0	0.07	0.23	1	18000	sdgamma[13,5]	1.12	1.27	0.01	0.17	0.56	1.7	4.49	1	27000
a[14,5]	0	0.12	-0.31	-0.13	-0.05	0.01	0.16	1	8100	sdgamma[14,5]	1.17	1.28	0.02	0.19	0.62	1.77	4.52	1	5500
a[15,5]	0	0.06	-0.15	-0.06	-0.02	0.01	0.1	1	6300	sdgamma[15,5]	1	1.23	0.01	0.12	0.43	1.44	4.4	1	25000
a[1,6]	-0.05	0.06	-0.17	-0.09	-0.05	-0.01	0.05	1	640	sdgamma[1,6]	1.05	1.25	0.01	0.14	0.48	1.54	4.45	1	9000
a[2,6]	0.08	0.09	-0.09	0.02	0.08	0.15	0.27	1	2900	sdgamma[2,6]	1.17	1.28	0.02	0.2	0.63	1.77	4.53	1	27000
a[3,6]	0	0.09	-0.14	-0.03	0.02	0.08	0.21	1.01	430	sdgamma[3,6]	1.08	1.25	0.01	0.15	0.52	1.61	4.46	1	17000
a[4,6]	-0.09	0.07	-0.23	-0.13	-0.08	-0.04	0.03	1	880	sdgamma[4,6]	1.15	1.28	0.02	0.19	0.6	1.72	4.53	1	26000
a[5,6]	0	0.07	-0.14	-0.03	0.01	0.06	0.16	1	2000	sdgamma[5,6]	1.03	1.25	0.01	0.13	0.45	1.53	4.43	1	14000
a[6,6]	0	0.07	-0.18	-0.08	-0.03	0.01	0.09	1	530	sdgamma[6,6]	1.03	1.25	0.01	0.13	0.46	1.5	4.44	1	27000
a[7,6]	0	0.07	-0.15	-0.04	0	0.05	0.14	1	520	sdgamma[7,6]	1.03	1.25	0.01	0.13	0.45	1.51	4.44	1	22000
a[8,6]	0	0.08	-0.2	-0.1	-0.05	0	0.1	1	4200	sdgamma[8,6]	1.08	1.26	0.01	0.16	0.51	1.6	4.48	1	6100
a[9,6]	0	0.09	-0.12	0	0.06	0.12	0.25	1	3700	sdgamma[9,6]	1.12	1.27	0.01	0.17	0.57	1.68	4.47	1	9400
a[10,6]	0	0.09	-0.19	-0.08	-0.02	0.03	0.16	1	15000	sdgamma[10,6]	1.09	1.27	0.01	0.15	0.53	1.64	4.49	1	27000
a[11,6]	0	0.08	-0.23	-0.09	-0.03	0.01	0.11	1	1200	sdgamma[11,6]	1.07	1.26	0.01	0.14	0.5	1.61	4.46	1	4600
a[12,6]	0	0.11	-0.3	-0.13	-0.05	0	0.11	1	1800	sdgamma[12,6]	1.14	1.27	0.01	0.18	0.58	1.73	4.49	1	15000
a[13,6]	0	0.1	-0.19	-0.04	0.01	0.08	0.19	1	11000	sdgamma[13,6]	1.1	1.27	0.01	0.16	0.52	1.67	4.44	1	6300
a[14,6]	0	0.09	-0.11	0.01	0.07	0.13	0.27	1	7500	sdgamma[14,6]	1.16	1.28	0.02	0.19	0.6	1.75	4.49	1	27000
a[15,6]	0.08	0.06	-0.03	0.04	0.08	0.12	0.19	1	540	sdgamma[15,6]	1.15	1.28	0.02	0.19	0.6	1.74	4.49	1	9600
