# Supplementary material for: CoDaLoMic: An R package for modeling microbiome compositional and longitudinal data
Source: PLoS Comput Biol. 2026 Jun 22;22(6):e1014328. doi: 10.1371/journal.pcbi.1014328 (PMC13362355; doi:10.1371/journal.pcbi.1014328)
Supplement: S8 Table — BPBM. Estimation quality. Parameter information after obtaining the parameters of the BPBM model using MCMC. Due to the high quantity of parameters, the information for all the parameters is in two tables, S7 and S8 Tables. The parameters with a mean of zero, but non-zero values for the standard deviation and quantiles, are those whose credible intervals include zero at the center. The StudyingParam function has adjusted their mean to zero. Since the estimated Rhat is less than 1.1 and the effective sample size (n.eff) exceeds 100, the quality of the estimation can be considered satisfactory. (PDF) [file pcbi.1014328.s008.pdf]

Table S8. Cockroach dataset. BPBM. Estimation quality. Parameter information after obtaining the parameters of the BPBM model using MCMC. Due to the high quantity of parameters, the information for all the parameters is in two tables, Table S7 and Table S8. The parameters with a mean of zero, but non-zero values for the standard deviation and quantiles, are those whose credible intervals include zero at the center. The `StudyingParam` function has adjusted their mean to zero. Since the estimated Rhat is less than 1.1 and the effective sample size (n.eff) exceeds 100, the quality of the estimation can be considered satisfactory.

	mean	sd	2.5%	25%	50%	75%	97.5%	Rhat	n.eff		mean	sd	2.5%	25%	50%	75%	97.5%	Rhat	n.eff
a[1,7]	0.08	0.07	-0.06	0.02	0.08	0.13	0.23	1	8100	sdgamma[1,7]	1.14	1.28	0.02	0.18	0.57	1.7	4.53	1	27000
a[2,7]	0.18	0.12	-0.04	0.09	0.18	0.26	0.43	1	6000	sdgamma[2,7]	1.37	1.31	0.04	0.33	0.86	2.08	4.58	1	27000
a[3,7]	-0.27	0.12	-0.49	-0.35	-0.27	-0.19	-0.03	1	6300	sdgamma[3,7]	1.53	1.32	0.11	0.46	1.07	2.33	4.65	1	27000
a[4,7]	0.15	0.09	-0.01	0.09	0.15	0.2	0.32	1	19000	sdgamma[4,7]	1.3	1.29	0.04	0.29	0.79	1.98	4.56	1	27000
a[5,7]	0.09	0.1	-0.09	0.01	0.08	0.15	0.29	1	19000	sdgamma[5,7]	1.18	1.29	0.02	0.2	0.62	1.79	4.53	1	27000
a[6,7]	0.12	0.1	-0.06	0.05	0.11	0.18	0.31	1	3500	sdgamma[6,7]	1.24	1.29	0.02	0.24	0.7	1.9	4.53	1	27000
a[7,7]	0	0.09	-0.15	-0.04	0.01	0.07	0.2	1	16000	sdgamma[7,7]	1.05	1.25	0.01	0.14	0.48	1.53	4.44	1	3000
a[8,7]	0.11	0.11	-0.09	0.03	0.1	0.18	0.34	1	9100	sdgamma[8,7]	1.22	1.29	0.02	0.22	0.68	1.86	4.53	1	27000
a[9,7]	-0.12	0.14	-0.4	-0.21	-0.11	-0.02	0.13	1	27000	sdgamma[9,7]	1.27	1.3	0.02	0.26	0.74	1.95	4.56	1	27000
a[10,7]	0.13	0.11	-0.08	0.04	0.12	0.2	0.36	1	27000	sdgamma[10,7]	1.26	1.29	0.03	0.26	0.73	1.93	4.53	1	18000
a[11,7]	0.17	0.13	-0.06	0.07	0.16	0.25	0.43	1	14000	sdgamma[11,7]	1.33	1.3	0.03	0.3	0.83	2.03	4.57	1	9200
a[12,7]	0	0.13	-0.16	-0.01	0.07	0.16	0.35	1	27000	sdgamma[12,7]	1.2	1.29	0.02	0.21	0.65	1.83	4.5	1	27000
a[13,7]	0	0.14	-0.18	-0.02	0.06	0.16	0.35	1	27000	sdgamma[13,7]	1.19	1.28	0.02	0.21	0.65	1.82	4.52	1	27000
a[14,7]	0	0.13	-0.23	-0.06	0.01	0.09	0.28	1	18000	sdgamma[14,7]	1.14	1.27	0.02	0.18	0.59	1.73	4.46	1	27000
a[15,7]	0	0.08	-0.19	-0.09	-0.04	0.01	0.11	1	4300	sdgamma[15,7]	1.06	1.25	0.01	0.14	0.49	1.57	4.43	1	11000
a[1,8]	0.13	0.08	-0.02	0.07	0.12	0.18	0.3	1	7600	sdgamma[1,8]	1.26	1.28	0.04	0.26	0.73	1.92	4.55	1	15000
a[2,8]	0	0.1	-0.24	-0.1	-0.03	0.02	0.15	1	27000	sdgamma[2,8]	1.11	1.28	0.01	0.16	0.55	1.67	4.48	1	10000
a[3,8]	0	0.11	-0.24	-0.09	-0.02	0.04	0.18	1	27000	sdgamma[3,8]	1.12	1.28	0.01	0.16	0.55	1.69	4.49	1	27000
a[4,8]	0.07	0.08	-0.08	0.01	0.06	0.12	0.24	1	11000	sdgamma[4,8]	1.11	1.27	0.01	0.16	0.56	1.68	4.45	1	27000
a[5,8]	0	0.09	-0.11	-0.01	0.04	0.11	0.24	1	16000	sdgamma[5,8]	1.11	1.26	0.01	0.16	0.54	1.65	4.45	1	25000
a[6,8]	0	0.08	-0.1	0	0.05	0.11	0.23	1	27000	sdgamma[6,8]	1.09	1.26	0.01	0.15	0.52	1.64	4.45	1	16000
a[7,8]	0.13	0.1	-0.05	0.06	0.12	0.19	0.32	1	27000	sdgamma[7,8]	1.27	1.3	0.03	0.26	0.73	1.96	4.57	1	2300
a[8,8]	0	0.1	-0.13	0	0.05	0.13	0.27	1	27000	sdgamma[8,8]	1.15	1.28	0.02	0.18	0.59	1.73	4.52	1	27000
a[9,8]	0	0.13	-0.33	-0.14	-0.05	0.02	0.18	1	17000	sdgamma[9,8]	1.18	1.29	0.02	0.2	0.63	1.79	4.52	1	17000
a[10,8]	0	0.1	-0.14	-0.01	0.04	0.12	0.26	1	3600	sdgamma[10,8]	1.12	1.27	0.02	0.17	0.56	1.69	4.51	1	13000
a[11,8]	0.12	0.12	-0.11	0.02	0.11	0.2	0.36	1	27000	sdgamma[11,8]	1.25	1.29	0.02	0.25	0.72	1.9	4.53	1	6600
a[12,8]	-0.15	0.14	-0.44	-0.25	-0.15	-0.05	0.09	1	27000	sdgamma[12,8]	1.32	1.3	0.03	0.29	0.81	2.02	4.58	1	27000
a[13,8]	0	0.13	-0.19	-0.02	0.05	0.14	0.32	1	27000	sdgamma[13,8]	1.21	1.3	0.02	0.21	0.66	1.87	4.56	1	7100
a[14,8]	0	0.13	-0.34	-0.15	-0.05	0.02	0.18	1	14000	sdgamma[14,8]	1.19	1.29	0.02	0.21	0.64	1.8	4.54	1	25000
a[15,8]	0	0.07	-0.12	-0.04	0	0.05	0.15	1	20000	sdgamma[15,8]	1.01	1.24	0.01	0.12	0.43	1.46	4.42	1	15000
a[1,9]	0.13	0.09	-0.03	0.07	0.13	0.2	0.32	1	27000	sdgamma[1,9]	1.26	1.29	0.03	0.27	0.74	1.9	4.55	1	27000
a[2,9]	0.19	0.13	-0.04	0.1	0.19	0.28	0.45	1	27000	sdgamma[2,9]	1.39	1.32	0.05	0.34	0.88	2.13	4.61	1	27000
a[3,9]	0	0.12	-0.22	-0.06	0.01	0.08	0.25	1	3600	sdgamma[3,9]	1.13	1.28	0.01	0.17	0.56	1.71	4.5	1	27000
a[4,9]	0.2	0.1	0.01	0.13	0.2	0.26	0.39	1	6300	sdgamma[4,9]	1.4	1.3	0.08	0.36	0.9	2.1	4.61	1	13000
a[5,9]	0	0.1	-0.19	-0.05	0.01	0.07	0.22	1	27000	sdgamma[5,9]	1.08	1.26	0.01	0.15	0.51	1.62	4.46	1	27000
a[6,9]	0	0.09	-0.15	-0.03	0.02	0.08	0.22	1	6300	sdgamma[6,9]	1.08	1.26	0.01	0.15	0.51	1.6	4.45	1	22000
a[7,9]	0	0.1	-0.16	-0.03	0.03	0.1	0.25	1	16000	sdgamma[7,9]	1.13	1.27	0.01	0.17	0.56	1.71	4.48	1	5600
a[8,9]	0	0.12	-0.16	-0.01	0.05	0.13	0.31	1	27000	sdgamma[8,9]	1.16	1.28	0.02	0.19	0.61	1.76	4.5	1	27000
a[9,9]	0	0.14	-0.24	-0.06	0.02	0.11	0.31	1	27000	sdgamma[9,9]	1.17	1.28	0.02	0.2	0.63	1.76	4.49	1	16000
a[10,9]	0.13	0.13	-0.1	0.03	0.12	0.22	0.41	1	27000	sdgamma[10,9]	1.27	1.29	0.02	0.26	0.76	1.95	4.54	1	27000
a[11,9]	0	0.13	-0.22	-0.04	0.03	0.12	0.31	1	10000	sdgamma[11,9]	1.16	1.28	0.02	0.19	0.6	1.75	4.52	1	12000
a[12,9]	0	0.14	-0.26	-0.06	0.02	0.11	0.32	1	13000	sdgamma[12,9]	1.19	1.29	0.02	0.2	0.63	1.8	4.52	1	23000
a[13,9]	0	0.15	-0.31	-0.1	-0.01	0.07	0.29	1	27000	sdgamma[13,9]	1.18	1.29	0.02	0.2	0.63	1.81	4.51	1	22000
a[14,9]	0	0.15	-0.21	-0.02	0.06	0.17	0.39	1	14000	sdgamma[14,9]	1.23	1.3	0.02	0.23	0.68	1.87	4.55	1	27000
a[15,9]	0.13	0.08	-0.02	0.07	0.13	0.19	0.3	1	4400	sdgamma[15,9]	1.27	1.29	0.04	0.26	0.75	1.93	4.56	1	20000
a[1,10]	0.13	0.09	-0.02	0.06	0.12	0.18	0.31	1.01	370	sdgamma[1,10]	1.25	1.29	0.03	0.25	0.72	1.9	4.55	1	4000
a[2,10]	0	0.13	-0.18	-0.02	0.04	0.13	0.32	1	720	sdgamma[2,10]	1.15	1.27	0.02	0.19	0.61	1.74	4.49	1	10000
a[3,10]	0.1	0.12	-0.11	0.01	0.09	0.18	0.34	1	810	sdgamma[3,10]	1.22	1.29	0.02	0.22	0.68	1.85	4.54	1	3700
a[4,10]	0.13	0.09	-0.03	0.06	0.12	0.19	0.31	1.01	420	sdgamma[4,10]	1.25	1.29	0.03	0.25	0.71	1.9	4.54	1	4900
a[5,10]	0.11	0.11	-0.07	0.04	0.11	0.18	0.34	1	640	sdgamma[5,10]	1.23	1.28	0.02	0.24	0.71	1.86	4.54	1	7700
a[6,10]	0.18	0.11	-0.02	0.1	0.18	0.26	0.41	1.01	490	sdgamma[6,10]	1.36	1.3	0.05	0.33	0.86	2.08	4.56	1	2300
a[7,10]	0.15	0.11	-0.04	0.07	0.15	0.23	0.38	1.01	430	sdgamma[7,10]	1.3	1.29	0.03	0.29	0.79	1.97	4.54	1	6500
a[8,10]	0.18	0.12	-0.04	0.09	0.18	0.26	0.42	1	1900	sdgamma[8,10]	1.36	1.3	0.04	0.33	0.87	2.08	4.58	1	15000
a[9,10]	0	0.14	-0.21	-0.03	0.04	0.14	0.34	1	2400	sdgamma[9,10]	1.19	1.29	0.02	0.21	0.65	1.8	4.53	1	27000
a[10,10]	0	0.11	-0.15	-0.01	0.05	0.14	0.3	1	1200	sdgamma[10,10]	1.17	1.29	0.02	0.19	0.61	1.78	4.53	1	16000
a[11,10]	0.19	0.14	-0.06	0.09	0.19	0.28	0.47	1	1400	sdgamma[11,10]	1.38	1.3	0.04	0.34	0.9	2.12	4.56	1	11000
a[12,10]	0	0.14	-0.19	-0.02	0.05	0.15	0.36	1	2100	sdgamma[12,10]	1.21	1.3	0.02	0.21	0.66	1.88	4.54	1	27000
a[13,10]	0	0.14	-0.36	-0.16	-0.06	0.02	0.2	1	5000	sdgamma[13,10]	1.22	1.3	0.02	0.22	0.67	1.87	4.56	1	27000
a[14,10]	0	0.13	-0.25	-0.07	0.01	0.09	0.28	1	3000	sdgamma[14,10]	1.15	1.28	0.02	0.19	0.6	1.75	4.51	1	27000
a[15,10]	0.12	0.09	-0.03	0.05	0.12	0.18	0.31	1.01	340	sdgamma[15,10]	1.25	1.3	0.03	0.25	0.71	1.92	4.55	1	12000
