# Supplementary material for: CoDaLoMic: An R package for modeling microbiome compositional and longitudinal data
Source: PLoS Comput Biol. 2026 Jun 22;22(6):e1014328. doi: 10.1371/journal.pcbi.1014328 (PMC13362355; doi:10.1371/journal.pcbi.1014328)

Fig S1. Results obtained with FBM in cockroach dataset.A. Temporal representation of taxa across all time points. B. Principal Component Analysis (PCA) of the estimated parameters, enabling visualization of bacterial taxa with similar dynamics; taxa positioned closer together in the PCA space exhibit more similar behavior. C. Variance over time.

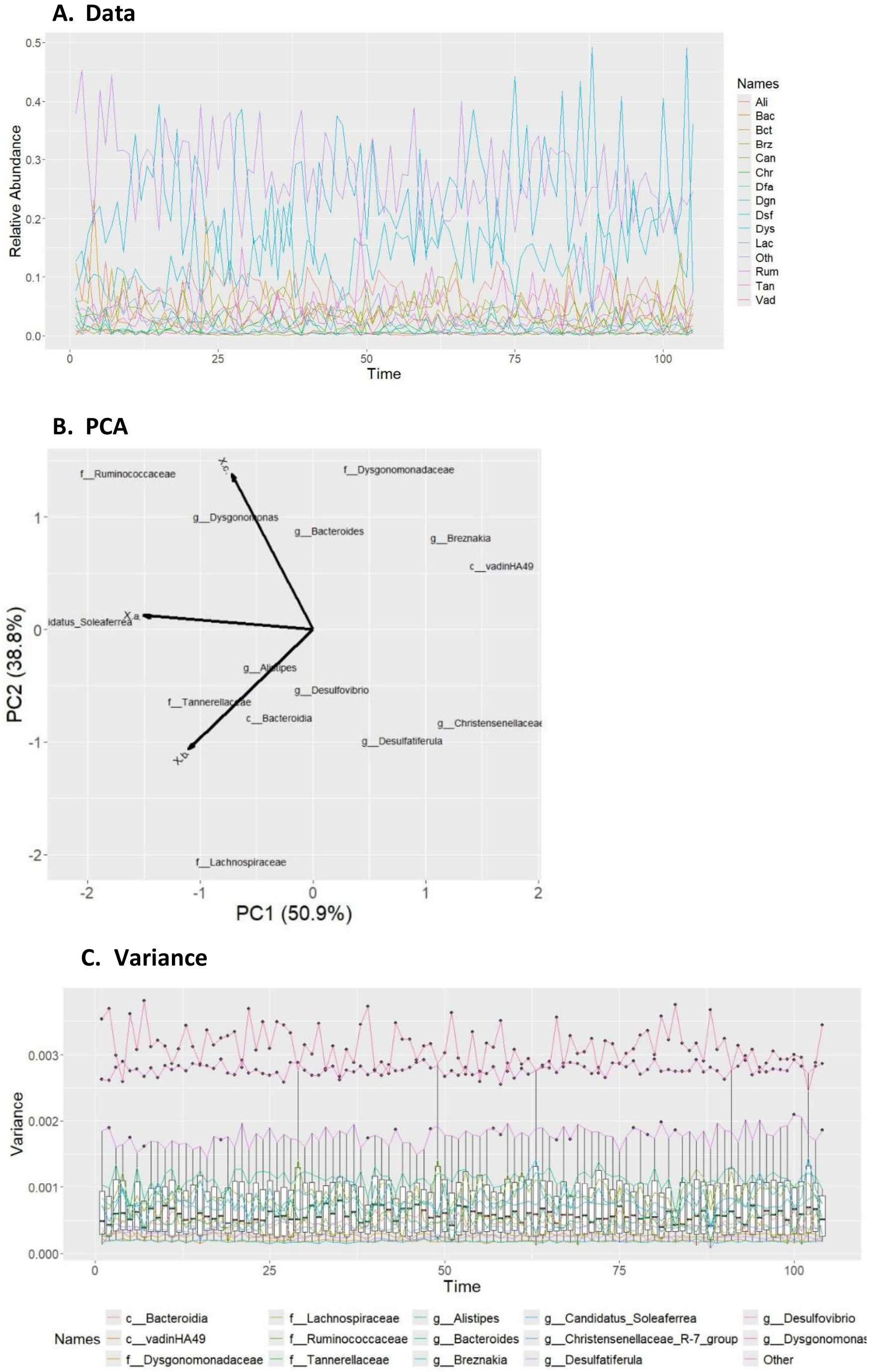

Supplement: S1 Fig — A. Temporal representation of taxa across all time points. B. Principal Component Analysis (PCA) of the estimated parameters, enabling visualization of bacterial taxa with similar dynamics; taxa positioned closer together in the PCA space exhibit more similar behavior. C. Variance over time. (PDF) [file pcbi.1014328.s009.pdf]
