# Supplementary material for: CoDaLoMic: An R package for modeling microbiome compositional and longitudinal data
Source: PLoS Comput Biol. 2026 Jun 22;22(6):e1014328. doi: 10.1371/journal.pcbi.1014328 (PMC13362355; doi:10.1371/journal.pcbi.1014328)

Fig S2. Results obtained using seqtime in cockroach dataset. In panels A and D, red indicates negative interactions while green denotes positive ones. Panel B reveals that the simulated correlation is not higher than the lag-1 autocorrelation, suggesting that the interaction matrix contributes little beyond the inherent temporal inertia of the data. We assign a number to each bacterium for easier identification: 1 is g_Dysgonomonas, 2 is g_Bacteroides, 3 is f_Lachnospiraceae, 4 is g_Desulfovibrio, 5 is g_Candidatus_Soleaferrea, 6 is g_Alistipes, 7 is f_Ruminococcaceae, 8 is c_Bacteroidia, 9 is g_Breznakia, 10 is f_Tannerellaceae, 11 is g_Christensenellaceae_R-7_group, 12 is f_Dysgonomonadaceae, 13 is c_vadinHA49, 14 is g_Desulfatiferula, 15 is Other.

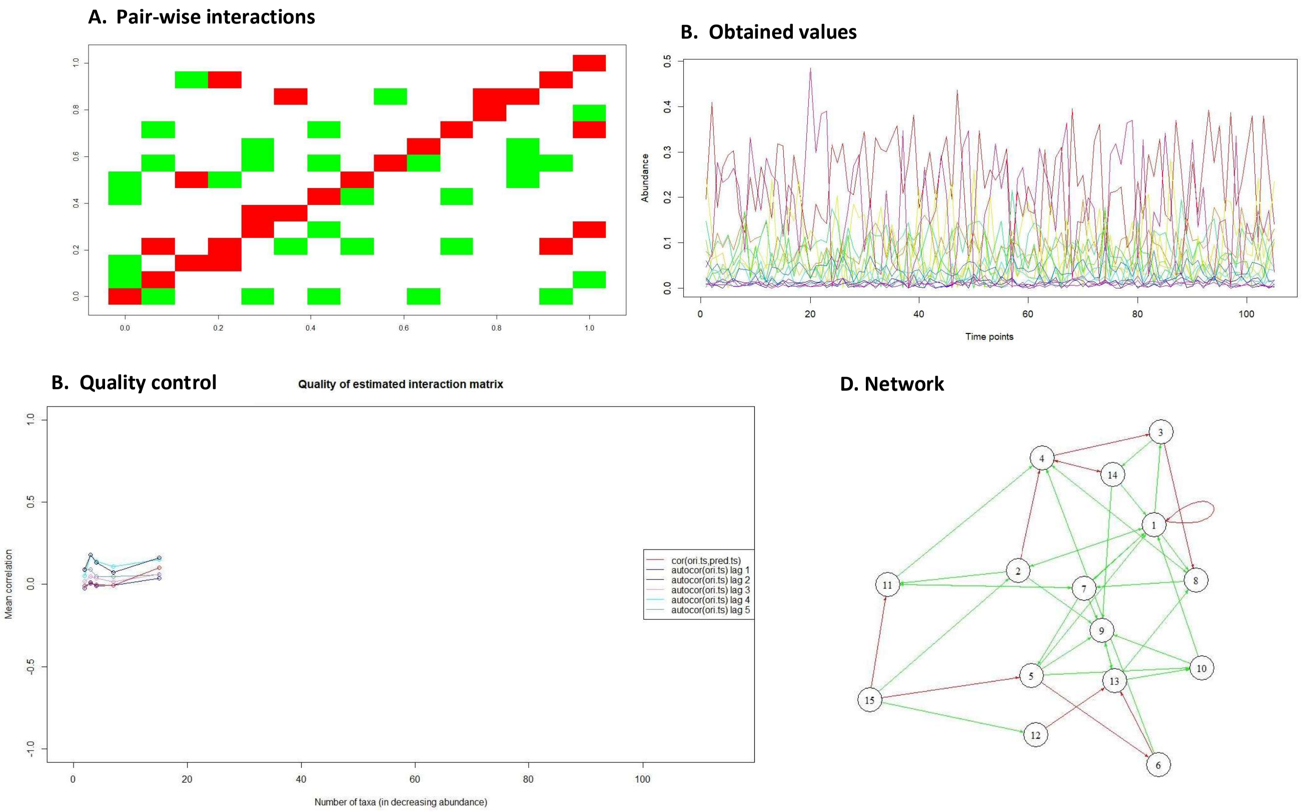

Supplement: S2 Fig — Results obtained using seqtime in cockroach dataset. In panels A and D, red indicates negative interactions while green denotes positive ones. Panel B reveals that the simulated correlation is not higher than the lag-1 autocorrelation, suggesting that the interaction matrix contributes little beyond the inherent temporal inertia of the data. We assign a number to each bacterium for easier identification: 1 is g_Dysgonomonas, 2 is g_Bacteroides, 3 is f_Lachnospiraceae, 4 is g_Desulfovibrio, 5 is g_Candidatus_Soleaferrea, 6 is g_Alistipes, 7 is f_Ruminococcaceae, 8 is c_Bacteroidia, 9 is g_Breznakia, 10 is f_Tannerellaceae, 11 is g_Christensenellaceae_R-7_group, 12 is f_Dysgonomonadaceae, 13 is c_vadinHA49, 14 is g_Desulfatiferula, 15 is Other. (PDF) [file pcbi.1014328.s010.pdf]
