# Supplementary material for: CoDaLoMic: An R package for modeling microbiome compositional and longitudinal data
Source: PLoS Comput Biol. 2026 Jun 22;22(6):e1014328. doi: 10.1371/journal.pcbi.1014328 (PMC13362355; doi:10.1371/journal.pcbi.1014328)

Fig S3. Results obtained with BiomeHorizon in cockroach dataset. Values are centered around a reference point. The plotting area is segmented into quartile bands extending above and below this origin. Darker blue bands represent progressively higher values above the origin, while darker red bands indicate increasingly lower values below it. Negative bands are symmetrically mirrored upward to enhance visual interpretation.

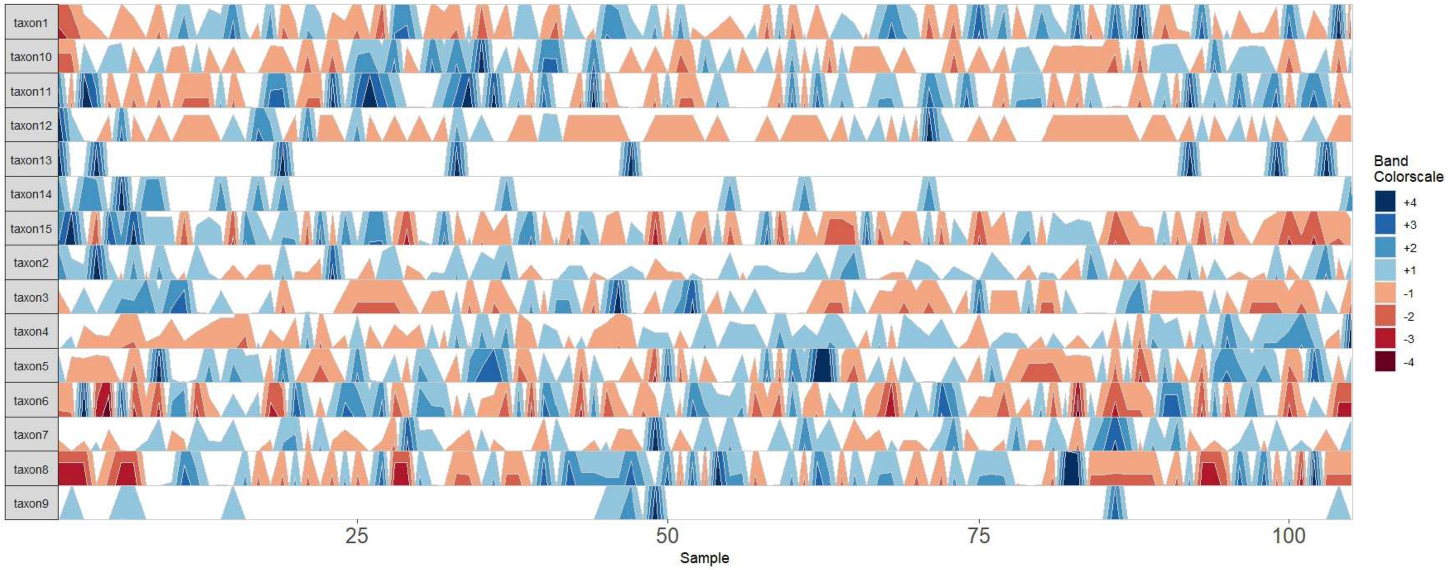

Supplement: S3 Fig — Results obtained with BiomeHorizon in cockroach dataset. Values are centered around a reference point. The plotting area is segmented into quartile bands extending above and below this origin. Darker blue bands represent progressively higher values above the origin, while darker red bands indicate increasingly lower values below it. Negative bands are symmetrically mirrored upward to enhance visual interpretation. (PDF) [file pcbi.1014328.s011.pdf]
