# Supplementary material for: CoDaLoMic: An R package for modeling microbiome compositional and longitudinal data
Source: PLoS Comput Biol. 2026 Jun 22;22(6):e1014328. doi: 10.1371/journal.pcbi.1014328 (PMC13362355; doi:10.1371/journal.pcbi.1014328)

Fig S4. Results obtained with BPBM in cockroach dataset. A: Dendrogram illustrating the Principal Balances. B: Temporal profile of the selected Principal Balances across all time points. Values closer to zero indicate greater similarity in the relationships between the groups within each balance. C: Variance of the taxa over time.

A. Dendogram

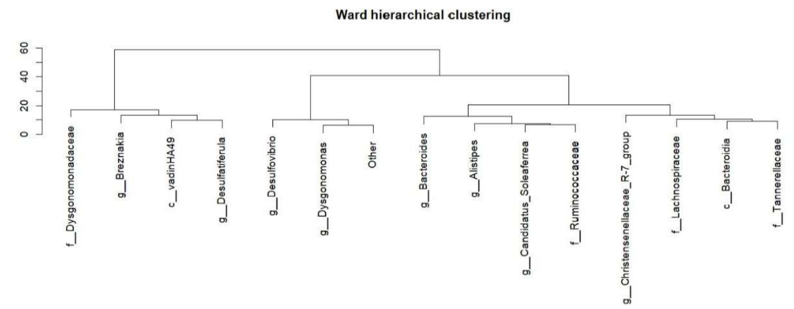

B. Selected Principal Balances

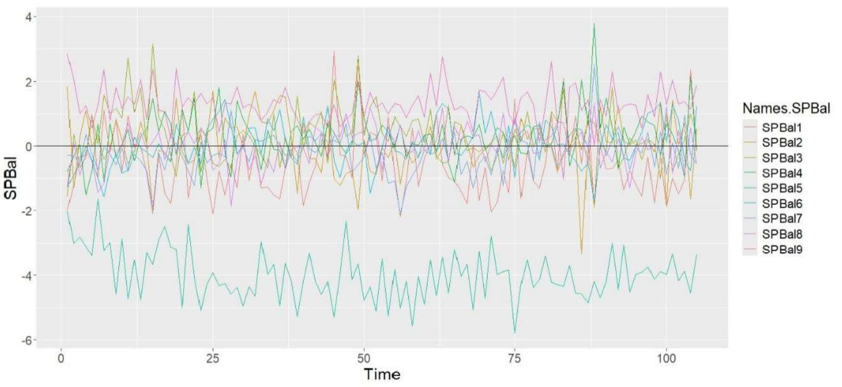

C. Variance

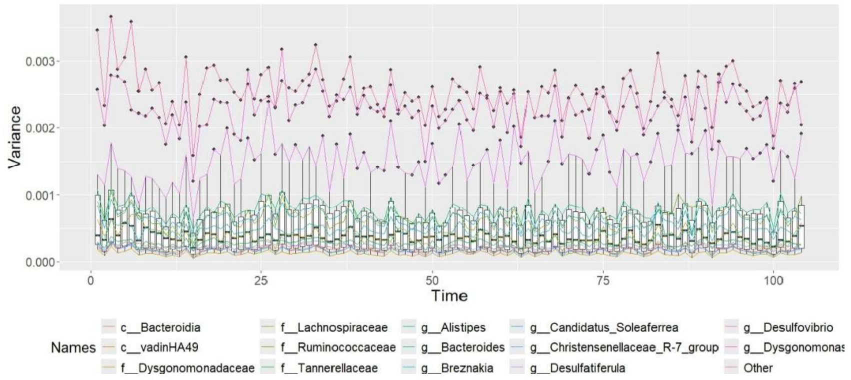

Supplement: S4 Fig — A: Dendrogram illustrating the Principal Balances. B: Temporal profile of the selected Principal Balances across all time points. Values closer to zero indicate greater similarity in the relationships between the groups within each balance. C: Variance of the taxa over time. (PDF) [file pcbi.1014328.s012.pdf]
