# Supplementary material for: CoDaLoMic: An R package for modeling microbiome compositional and longitudinal data
Source: PLoS Comput Biol. 2026 Jun 22;22(6):e1014328. doi: 10.1371/journal.pcbi.1014328 (PMC13362355; doi:10.1371/journal.pcbi.1014328)
Supplement: S5 Fig — The abbreviation Dys stands for g_Dysgonomonas, while Bct represents g_Bacteroides. These are followed by Lac, which corresponds to f_Lachnospiraceae, and Dsf, which refers to g_Desulfovibrio. Continuing on, Can is the abbreviation for g_Candidatus_Soleaferrea, and Ali represents g_Alistipes. Rum corresponds to f_Ruminococcaceae, whereas Bac refers to c_Bacteroidia. In the next set, Brz stands for g_Breznakia, and Tan refers to f_Tannerellaceae. Meanwhile, Chr represents g_Christensenellaceae_R7_group, and Dgn stands for f_Dysgonomonadaceae. Lastly, Vad corresponds to c_vadinHA49, and Dfa represents g_Desulfatiferula. The abbreviation Oth simply refers to Other. (PDF) [file pcbi.1014328.s013.pdf]

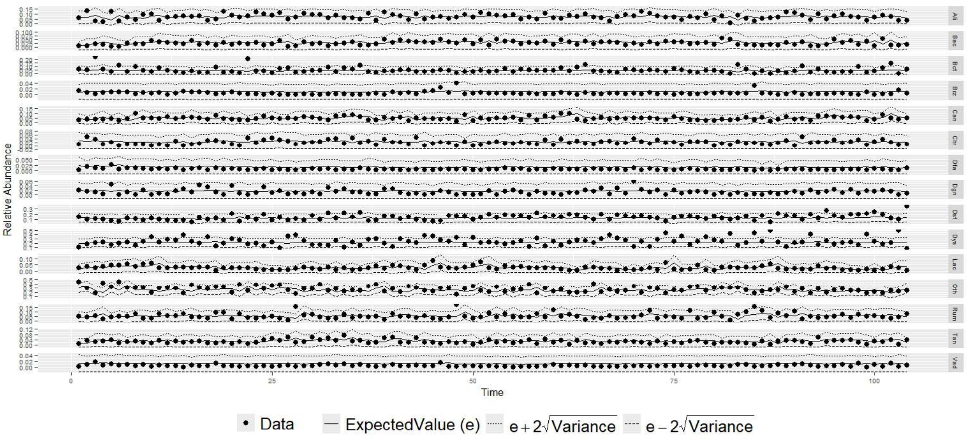

Fig S5. Expected values obtained with the FBM model. The abbreviation Dys stands for g_Dysgonomonas, while Bct represents g_Bacteroides. These are followed by Lac, which corresponds to f_Lachnospiraceae, and Dsf, which refers to g_Desulfovibrio. Continuing on, Can is the abbreviation for g_Candidatus_Soleaferrea, and Ali represents g_Alistipes. Rum corresponds to f_Ruminococcaceae, whereas Bac refers to c_Bacteroidia. In the next set, Brz stands for g_Breznakia, and Tan refers to f_Tannerellaceae. Meanwhile, Chr represents g_Christensenellaceae_R7_group, and Dgn stands for f_Dysgonomonadaceae. Lastly, Vad corresponds to c_vadinHA49, and Dfa represents g_Desulfatiferula. The abbreviation Oth simply refers to Other.
