# Supplementary material for: CoDaLoMic: An R package for modeling microbiome compositional and longitudinal data
Source: PLoS Comput Biol. 2026 Jun 22;22(6):e1014328. doi: 10.1371/journal.pcbi.1014328 (PMC13362355; doi:10.1371/journal.pcbi.1014328)
Supplement: S1 Appendix — A document that includes a detailed explanation and a pipeline of the preprocessing stage. (PDF) [file pcbi.1014328.s015.pdf]

Preprocessing, Quality control, Zero Imputation and Impact on Modeling

Irene Creus-Martí[1,*], Andrés Moya[2, 3, 4], Francisco J. Santonja[1]

[1] Department of Statistics and Operation Research, Universitat de València, Valencia, Spain
[2] Institute for Integrative Systems Biology (I2Sysbio), Universitat de València and CSIC, València, Spain
[3] The Foundation for the Promotion of Health and Biomedical Research of Valencia Region (FISABIO), Valencia, Spain
[4] CIBER in Epidemiology and Public Health (CIBERESP), Madrid, Spain

* irene.creus@uv.es

1 Preprocessing, Quality control, Zero Imputation and Impact on Modeling

The models implemented in CoDaLoMic are designed to analyze datasets in which each row sums to one. Microbiome datasets derived from high-throughput sequencing typically undergo several bioinformatic processing steps before relative abundances are obtained. While the model operates independently of the quality control and preprocessing procedures applied prior to modeling, the limitations inherent to these techniques must be carefully considered when interpreting the results. In addition, it is essential to document the choices done to facilitate reproducibility. Fig A presents the pipeline of the preprocessing stage.

Several software platforms are available to support the implementation of these methodological steps. QIIME 2 [5, 3], EasyAmplicon [18], Wekemo Bioincloud [10], iNAP 2 [24] and EasyMetagenome [2] provide comprehensive frameworks for microbiome data analysis, encompassing processes from data collection to statistical evaluation. Further details regarding these tools and guidance on their appropriate application can be found in [7].

We will now turn our attention to the specific details of the processes, beginning with Quality control (QC). QC encompasses the set of procedures and metrics designed to ensure that biological data generated through sequencing or other omics experiments are reliable, accurate, and suitable for

Fig A: Pipeline of the preprocessing stage.

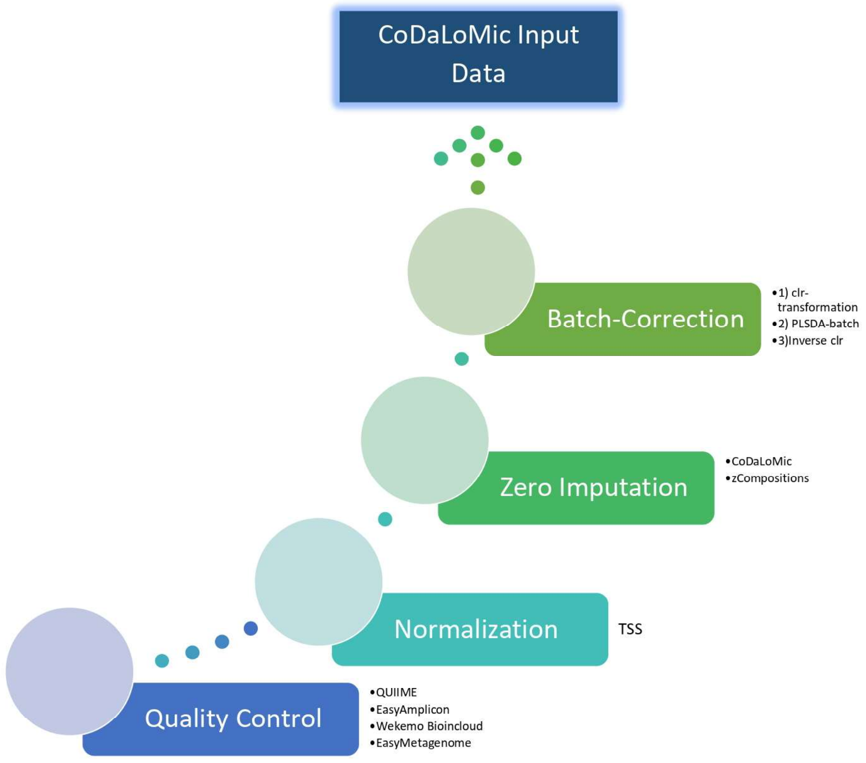

downstream analyses. Without rigorous quality control, the features extracted from a dataset may primarily reflect technical or experimental artifacts, such as batch effects introduced during sample preparation, rather than genuine biological responses to an exposure [6]. Quality control involves evaluating how to handle samples that fail to provide sufficient information, such as those characterized by low sequencing depth, poor alignment rates, reduced overlap fraction of reads in peaks, absence of the nucleosome-free region peak, absence of the mononucleosomal peak, low transcription start site enrichment, or a high percentage of PCR duplicates [6]. Guidance on appropriate actions in such cases can be found in [6]. In terms of feature and sample filtering, typical strategies involve excluding features (such as taxa) that occur in fewer than 10–20% of samples, removing taxa that display extremely low relative abundances (e.g., below 0.01%) across the dataset, or discarding taxa that contain zeros throughout the time series. Although these thresholds may be adjusted according to the specific study design and sequencing depth, their rationale and application must be explicitly reported.

If the dataset is composed of relative abundances, it can be directly subjected to zero imputation and/or batch correction. In other cases, normalization of the data is required prior to applying CoDaLoMic. There are several approaches to normalize microbiome datasets, such as rarefying, rarefaction, Variance Stabilizing Transformation (VST) [1], Trimmed Mean of M-values (TMM) [26] or Total Sum Scaling (TSS). Rarefying and rarefaction have similarities, rarefaction relies on random subsampling to equalize sequencing depth while rarefying is generally performed as a single iteration [4]. However, the use of subsampling has been increasingly questioned, as it leads to a reduction in information and a loss of precision [21, 11]. As it is explained in [11], VST and TMM are similar to the compositional approach but they prove less appropriate in datasets characterized by pronounced asymmetry or sparsity [9, 12]. TSS converts raw counts into relative abundances by dividing each feature by the total number of reads within a sample. A limitation of TSS is that it fails to account for heteroscedasticity under the assumption that counts follow a negative binomial distribution [21], which is not our case. The appropriate method for analyzing compositional data involves the application of ratio transformations [22]. Such transformations effectively capture the relational structure among features within the dataset and these ratios remain invariant whether the data are expressed as raw counts or as proportions [11]. By taking the logarithm of these ratios (log-ratios), the data are rendered symmetric, linearly related and situated within a log-ratio

coordinate space [22, 11]. All models implemented in CoDaLoMic rely on log-ratios. Consequently, employing TSS in conjunction with CoDaLoMic is appropriate, since the log-ratios derived from counts or proportions yield identical values.

It is well established that microbiome datasets have zero inflation [23]. CoDaLoMic contemplates the use of two ways to impute zeros (available at *zCompositions* package [22]: the multiplicative Kaplan-Meier smoothing spline replacement [22] and the multiplicative simple replacement [22, 20]. In both methods, a detection limit is decided and all the values under this detection limit are considered censored values. Talking about the performance of the imputation step, substituted values often impose artificial patterns that can obscure the original data [14]. To address this limitation, alternative methods such as Regression on Order Statistics (ROS) and the Kaplan–Meier estimator have been proposed [14]. No approach has consistently outperformed others, outcomes vary with testing conditions and performance measures [22]. Methods specifically adapted for compositional data are presented in [22] and preserve the relative structure of the dataset. This preservation is critical, as it respects the reliability of subsequent statistical analyses by maintaining the integrity of the underlying relationships among variables.

To simulate censored values effectively, the multiplicative Kaplan-Meier smoothing spline replacement [22] follows three key steps. First, random values are generated from a uniform distribution within the interval between zero and the detection limit. This ensures an unbiased representation of values that could exist below the threshold but remain undetected. Next, a cubic smoothing spline is applied to fit the empirical cumulative distribution function of the data. Finally, inverse transformation sampling is performed by mapping the previously generated uniform values onto the spline function. This step ensures that the simulated values conform to the estimated distribution structure, providing a realistic approximation of censored data. By following these steps, the method effectively reconstructs missing values while preserving the integrity of the dataset. The multiplicative simple replacement [22, 20] offers a structured alternative to the conventional practice of substituting censored values with a predetermined fraction of the threshold. The defining characteristic of this approach is that, after substitution, the remaining components are adjusted to maintain constant the value of the sum of the components.

Zero imputation in CoDaLoMic is performed through the `ZeroData` func-

tion. The dataset provided to the `ZeroData` function must adhere the following structure: the first column of the dataset must contain the time point information, while the remaining columns must represent the relative abundance of each bacterium. Consequently, the sum of each row (excluding the time point information) must equal one.

```
> head(cockroach[,c(1:5)])
       Time g__Dysgonomonas g__Fusobacterium g__Bacteroides f__Lachnospiraceae
K3a001    1      0.07560585       0.01632285     0.12357300         0.01477783
K3a002    2      0.10846380       0.15578647     0.06284101         0.02645793
K3a003    3      0.17318365       0.06258845     0.05532145         0.03536502
K3a004    4      0.20467225       0.05169049     0.23045835         0.02308526
K3a005    5      0.22032927       0.23473095     0.06045431         0.03816444
K3a006    6      0.15682989       0.09483659     0.02810507         0.04810291
```

The `ZeroData` function have three options. If we select `method="nozeros"` the bacteria that contain any zeros is removed and a new column called Other is introduced in the dataset. After removing the bacteria with zeros, this new column ensures that the sum of all the bacteria is equal to one at all time points. This column can be interpreted as the information for all less abundant bacteria. In order to replace the zeros we can use multiplicative Kaplan-Meier smoothing spline replacement [22] or the multiplicative simple replacement [22, 20], depending on whether we write `method="multKM"` or `method="multRepl"` respectively. Both methods use as detection limit the 0.01.

```
>DaTa=ZeroData(cockroach, "nozeros")
>head(DaTa[,c(1:5)])
       Time g__Dysgonomonas g__Bacteroides f__Lachnospiraceae g__Desulfovibrio
K3a001    1      0.07560585     0.12357300         0.01477783       0.12619095
K3a002    2      0.10846380     0.06284101         0.02645793       0.14410660
K3a003    3      0.17318365     0.05532145         0.03536502       0.08981188
K3a004    4      0.20467225     0.23045835         0.02308526       0.10464268
K3a005    5      0.22032927     0.06045431         0.03816444       0.10099175
K3a006    6      0.15682989     0.02810507         0.04810291       0.06992037
```

If you want to impute the values using another detection limit you can use directly the *zComposition* package implementing the following code and controlling the detection limit with the value introduced in `dl`. In the following example the detection limit is 0.00001. Note that if a row or column contains more than 80% of zeros, the function automatically removes that row or column and displays a message informing you of the deletion.

```
>DaTa_noZero_a=zCompositions::multKM(cockroach[,-1],label=0,  dl=rep(0.00001,ncol(cockroach[,-1])))

>DaTa_noZero_b=zCompositions::multRepl(cockroach[,-1], label=0,dl=rep(0.00001,ncol(cockroach[,-1])))
```

Batch effects represent systematic sources of variation that may arise from technical artefacts or uncontrolled biological covariates [30]. If left uncorrected, these effects introduce spurious variability and confounding factors

that can become aligned with the experimental groups under comparison [30]. Such distortions obscure genuine biological signals and substantially increase the risk of false discoveries, thereby undermining the validity and reproducibility of downstream analyses. [30] collets the methods used to face batch effects in different types of datasets: ComBat [29], SVA [16], Harmony [15], MNN [13], LINGER [28], PLSDA-batch [27], Limma [25], DESeq2 [19], Bayesian Dirichlet-multinomial regression [8] and ConQuR [17]. Among the methods considered, the only approach that explicitly accounts for the compositional nature of the data by applying a centered log-ratio (CLR) transformation prior to analysis is PLSDA-batch. For this reason, we regard PLSDA-batch as the most appropriate method for our dataset. Nevertheless, it is important to note that the inverse CLR transformation must subsequently be performed in order to recover the dataset for downstream analyses. Upon completion of these processes, we will obtain the dataset that can be used as input for the CoDaLoMic package.

References

[1] Simon Anders and Wolfgang Huber. Differential expression analysis for sequence count data. *Nature Precedings*, pages 1–1, 2010.

[2] Defeng Bai, Tong Chen, Jiani Xun, Chuang Ma, Hao Luo, Haifei Yang, Chen Cao, Xiaofeng Cao, Jianzhou Cui, Yuan-Ping Deng, et al. Easymetagenome: A user-friendly and flexible pipeline for shotgun metagenomic analysis in microbiome research. *iMeta*, page e70001, 2025.

[3] Evan Bolyen, Jai Ram Rideout, Matthew R Dillon, Nicholas A Bokulich, Christian C Abnet, Gabriel A Al-Ghalith, Harriet Alexander, Eric J Alm, Manimozhiyan Arumugam, Francesco Asnicar, et al. Reproducible, interactive, scalable and extensible microbiome data science using qiime 2. *Nature biotechnology*, 37(8):852–857, 2019.

[4] Ellen S Cameron, Philip J Schmidt, Benjamin J-M Tremblay, Monica B Emelko, and Kirsten M Müller. To rarefy or not to rarefy: Enhancing diversity analysis of microbial communities through next-generation sequencing and rarefying repeatedly. *BioRxiv*, pages 2020–09, 2020.

[5] J Gregory Caporaso, Justin Kuczynski, Jesse Stombaugh, Kyle Bittinger, Frederic D Bushman, Elizabeth K Costello, Noah Fierer, An-

tonio Gonzalez Peña, Julia K Goodrich, Jeffrey I Gordon, et al. Qiime allows analysis of high-throughput community sequencing data. *Nature methods*, 7(5):335–336, 2010.

[6] Arianna Comendul, Frederique Ruf-Zamojski, Colby T Ford, Pankaj Agarwal, Elena Zaslavsky, German Nudelman, Manoj Hariharan, Aliza Rubenstein, Hanna Pincas, Venugopalan D Nair, et al. Comprehensive guide for epigenetics and transcriptomics data quality control. *STAR protocols*, 6(1):103607, 2025.

[7] Irene Creus-Martí, Andrés Moya, and Francisco J Santonja. Methodology for microbiome data analysis: An overview. *Computers in Biology and Medicine*, 192:110157, 2025.

[8] Zhenwei Dai, Sunny H Wong, Jun Yu, and Yingying Wei. Batch effects correction for microbiome data with dirichlet-multinomial regression. *Bioinformatics*, 35(5):807–814, 2019.

[9] Andrew D Fernandes, Jean M Macklaim, Thomas G Linn, Gregor Reid, and Gregory B Gloor. Anova-like differential expression (aldex) analysis for mixed population rna-seq. *PloS one*, 8(7):e67019, 2013.

[10] Yunyun Gao, Guoxing Zhang, Shunyao Jiang, and Yong-Xin Liu. Wekemo bioincloud: A user-friendly platform for meta-omics data analyses. *Imeta*, 3(1):e175, 2024.

[11] G. B. Gloor, J. M. Macklaim, et al. Microbiome datasets are compositional: and this is not optional. *Frontiers in Microbiology*, 8, 2017.

[12] Gregory Brian Gloor, Jean M Macklaim, Michael Vu, and Andrew D Fernandes. Compositional uncertainty should not be ignored in high-throughput sequencing data analysis. *Austrian Journal of Statistics*, 45 (4):73–87, 2016.

[13] Laleh Haghverdi, Aaron TL Lun, Michael D Morgan, and John C Marioni. Batch effects in single-cell rna-sequencing data are corrected by matching mutual nearest neighbors. *Nature biotechnology*, 36(5):421–427, 2018.

[14] Dennis Helsel. Much ado about next to nothing: incorporating nondetects in science. *Annals of occupational hygiene*, 54(3):257–262, 2010.

[15] Ilya Korsunsky, Nghia Millard, Jean Fan, Kamil Slowikowski, Fan Zhang, Kevin Wei, Yuriy Baglaenko, Michael Brenner, Po-ru Loh, and Soumya Raychaudhuri. Fast, sensitive and accurate integration of single-cell data with harmony. *Nature methods*, 16(12):1289–1296, 2019.

[16] Jeffrey T Leek, W Evan Johnson, Hilary S Parker, Andrew E Jaffe, and John D Storey. The sva package for removing batch effects and other unwanted variation in high-throughput experiments. *Bioinformatics*, 28 (6):882–883, 2012.

[17] Wodan Ling, Jiuyao Lu, Ni Zhao, Anju Lulla, Anna M Plantinga, Weijia Fu, Angela Zhang, Hongjiao Liu, Hoseung Song, Zhigang Li, et al. Batch effects removal for microbiome data via conditional quantile regression. *Nature communications*, 13(1):5418, 2022.

[18] Yong-Xin Liu, Lei Chen, Tengfei Ma, Xiaofang Li, Maosheng Zheng, Xin Zhou, Liang Chen, Xubo Qian, Jiao Xi, Hongye Lu, et al. Easyamplicon: An easy-to-use, open-source, reproducible, and community-based pipeline for amplicon data analysis in microbiome research. *Imeta*, 2(1): e83, 2023.

[19] Michael I Love, Wolfgang Huber, and Simon Anders. Moderated estimation of fold change and dispersion for rna-seq data with deseq2. *Genome biology*, 15(12):550, 2014.

[20] Josep A Martín-Fernández, Carles Barceló-Vidal, and Vera Pawlowsky-Glahn. Dealing with zeros and missing values in compositional data sets using nonparametric imputation. *Mathematical Geology*, 35:253–278, 2003.

[21] Paul J McMurdie and Susan Holmes. Waste not, want not: why rarefying microbiome data is inadmissible. *PLoS computational biology*, 10 (4):e1003531, 2014.

[22] Javier Palarea-Albaladejo and Josep Antoni Martín-Fernández. zcompositions—r package for multivariate imputation of left-censored data under a compositional approach. *Chemometrics and Intelligent Laboratory Systems*, 143:85–96, 2015.

[23] Amy Y Pan. Statistical analysis of microbiome data: the challenge of sparsity. *Current Opinion in Endocrine and Metabolic Research*, 19: 35–40, 2021.

[24] Xi Peng, Kai Feng, Xingsheng Yang, Qing He, Bo Zhao, Tong Li, Shang Wang, and Ye Deng. inap 2.0: Harnessing metabolic complementarity in microbial network analysis. *Imeta*, 3(5):e235, 2024.

[25] Matthew E Ritchie, Belinda Phipson, DI Wu, Yifang Hu, Charity W Law, Wei Shi, and Gordon K Smyth. limma powers differential expression analyses for rna-sequencing and microarray studies. *Nucleic acids research*, 43(7):e47–e47, 2015.

[26] Mark D Robinson and Alicia Oshlack. A scaling normalization method for differential expression analysis of rna-seq data. *Genome biology*, 11 (3):R25, 2010.

[27] Yiwen Wang and Kim-Anh Lê Cao. Plsda-batch: a multivariate framework to correct for batch effects in microbiome data. *Briefings in Bioinformatics*, 24(2):bbac622, 2023.

[28] Joshua D Welch, Velina Kozareva, Ashley Ferreira, Charles Vanderburg, Carly Martin, and Evan Z Macosko. Single-cell multi-omic integration compares and contrasts features of brain cell identity. *Cell*, 177(7):1873–1887, 2019.

[29] Yuqing Zhang, Giovanni Parmigiani, and W Evan Johnson. Combat-seq: batch effect adjustment for rna-seq count data. *NAR genomics and bioinformatics*, 2(3):lqaa078, 2020.

[30] Ruwen Zhou, Siu Kin Ng, Joseph Jao Yiu Sung, Wilson Wen Bin Goh, and Sunny Hei Wong. Data pre-processing for analyzing microbiome data–a mini review. *Computational and Structural Biotechnology Journal*, 21:4804–4815, 2023.
