# Supplementary material for: CoDaLoMic: An R package for modeling microbiome compositional and longitudinal data
Source: PLoS Comput Biol. 2026 Jun 22;22(6):e1014328. doi: 10.1371/journal.pcbi.1014328 (PMC13362355; doi:10.1371/journal.pcbi.1014328)
Supplement: S2 Appendix — Document detailing the three models implemented in CoDaLoMic (Dirich-gLV, FBM, and BPBM). (PDF) [file pcbi.1014328.s016.pdf]

Models Implemented in CoDaLoMic

Irene Creus-Martí[1,*], Andrés Moya[2, 3, 4], Francisco J. Santonja[1]

1 Department of Statistics and Operation Research, Universitat de València, Valencia, Spain
2 Institute for Integrative Systems Biology (I2Sysbio), Universitat de València and CSIC, València, Spain
3 The Foundation for the Promotion of Health and Biomedical Research of Valencia Region (FISABIO), Valencia, Spain
4 CIBER in Epidemiology and Public Health (CIBERESP), Madrid, Spain

* irene.creus@uv.es

1 Compositional CoDa Basic Principles

One of the approaches used to analyze compositional data is the use of log-ratio transformations that project the Simplex (the sample space of compositional data) to real space [1]. We denote with $\mathbf{x} = (x_1, x_2, \ldots, x_D)$ a composition with D components and we denote with $g(x)$ the geometric mean of the composition. The additive log-ratio transformation (alr) and centered log-ratio transformation (clr) defined by [1] have the following expressions:

$$\mathrm{alr}(\mathbf{x}) = \left(\ln\left(\frac{x_1}{x_D}\right), \ln\left(\frac{x_2}{x_D}\right), \ldots, \ln\left(\frac{x_{D-1}}{x_D}\right)\right)$$

$$\mathrm{clr}(\mathbf{x}) = \left(\ln\left(\frac{x_1}{g(X)}\right), \ln\left(\frac{x_2}{g(X)}\right), \ldots, \ln\left(\frac{x_{D-1}}{g(X)}\right)\right)$$

The log-transformations were developed to analyze the relationship between components in a composition. The technique used with this objective is called balance [5]. A balance is a logarithmic transformation that compares two groups of components (parts of a composition), with the aim of capturing relevant contrasts between them. Using the notation applied by [9], balances have the following expression:

$$b = \sqrt{\frac{r \cdot s}{r+s}} \cdot \ln\left(\frac{g(\mathbf{x}_r)}{g(\mathbf{x}_s)}\right) \tag{1}$$

$$g(\mathbf{x}_r) = \exp(K) \cdot g(\mathbf{x}_s) \text{ with } K = \frac{b}{\sqrt{\frac{r \cdot s}{r+s}}} \tag{2}$$

where $\mathbf{x}_r$ and $\mathbf{x}_s$ are two non-overlapping groups of parts of a D-composition; r and s are the number of parts in $\mathbf{x}_r$ and $\mathbf{x}_s$ respectively. Operating with (1) we can obtain (2), where we can see that when the balance is near zero, the geometric means of the two groups are similar. As a result, on average, the abundance of the group at the numerator and the group at the denominator is similar. However, the greater the absolute value of the balance, the larger the difference between the geometric means, and consequently, on average, the abundance of the group at the numerator and the group at the denominator is different. Another compositional technique is Principal Balances. Principal balances are a specific selection of balances that maximize the explained variance in the transformed data; they function as the equivalent of principal components in PCA, but adapted for compositional data. It involves selecting the components of $\mathbf{x}_r$ and $\mathbf{x}_s$ that maximize the variance of the balance. This is because balances with greater variability will better explain the variability of the microbiome data [9]. They reduce the dimension with a minimum loss of information so they make it possible to estimate the reduction of parameters.

2 Models

In this package, the following models have been implemented. In summary, Table A presents the parameters of each model along with their interpretation.

• **Dirich-gLV**. We call the model defined in [2] the Dirich-gLV model. Let $\boldsymbol{y_t} = (y_{1t}, y_{2t}, \ldots, y_{Dt})$ be the vector that contains the relative abundance of all the taxa at time point t. Let D be the number of bacterial taxa present in the dataset. The Dirich-gLV model proposes that $\boldsymbol{y_t} \sim \text{Dirichlet}(\boldsymbol{\alpha_t})\ \forall t$ and $\tau = \sum_{i=1}^{D} \alpha_{it}\ \forall t$. In addition, with $i = 1, \ldots, D-1$, the model proposes the following regression.

$$\text{alr}(\alpha_{it}) = r_i \,\text{alr}(y_{i,t-1}) + \text{alr}(y_{i,t-1}) \cdot \Big[a_{i1} \,\text{alr}(y_{1,t-1}) + \cdots + a_{i,D-1} \,\text{alr}(y_{D-1,t-1})\Big] \tag{3}$$

Table A: Parameters interpretation. This table presents the model parameters, the information conveyed by each parameter and guidance on how to interpret their values.

**Model**	**Parameters**	**Parameters information**	**Interpreting the value**
Dirich-gLV	r_i in Equation 3	The capacity that each bacterium has to influence its own future abundance	The greater the absolute value, the stronger the influence (and vice versa).
	a_{ij} in Equation 3	Pair-wise interaction between bacteria	
FBM	a_{i1} in Equation 4	Each taxon's baseline abundance level	The greater the value, the stronger the influence (and vice versa)
	a_{i2} in Equation 4	Each taxon influence in its own presence over time	The greater the absolute value, the stronger the influence (and vice versa).
	a_{i3} in Equation 4)	The influence exerted by the surrounding taxa on the focal taxon.	
BPBM	a_{i0} in Equation 5)	Each taxon's baseline abundance level	The greater the value, the stronger the influence (and vice versa)
	a_{ij} in Equation 5) with $j \neq 0$	How the relationship between groups of taxa influence each bacteria.	The greater the absolute value, the stronger the influence (and vice versa).

Maximum likelihood estimation is carried out. The parameters are τ, r_i, $a_{i1}, \ldots, a_{i,D-1}$ with $i = 1, \ldots, D-1$. Note that working with equation (3) we can obtain the value of the Dirichlet parameters and calculate the expected value using $E[y_{it}] = \alpha_{it}/(\sum_1^D \alpha_{it})$. Regarding the interpretation of the parameters, the parameter r_i represents the self-influence or inertia of taxon i, reflecting the extent to which its current state predicts its own abundance at the subsequent time point. A high value of r_i suggests that the taxon exhibits stable and autocorrelated temporal dynamics. The parameters a_{ij} capture the interactions between taxa; specifically, the coefficient a_{ij} quantifies the effect that the abundance of taxon j at the previous time point has on the future abundance of taxon i. The greater the absolute value of the parameter, the stronger the influence on the taxa. Thus, these parameters characterize the dynamic ecological relationships among different bacteria within the community.

• **FBM**. We call the model defined in [3] FBM. We have introduced the FBM model considering just one previous point in time. In other words, we take into account the case when $P = 1$ following the notation in [3]. We denote $\boldsymbol{y_t} = (y_{1t}, y_{2t}, \ldots, y_{Dt})$, where y_{it} denotes the relative abundance of taxon i at time t and $i = 1, ..., D$. As in the Dirich-gLV model, $\boldsymbol{y_t} \sim$ Dirichlet$(\boldsymbol{\alpha_t})$, $\forall t$ and $\tau = \sum_{i=i}^D \alpha_{it}$, $\forall t$. This model proposes for $i = 1, \ldots, D-1$ the following regression.

$$\ln\left(\frac{\alpha_{it}}{\alpha_{Dt}}\right) = \mu_{it} = a_{i1} + a_{i2} \cdot \ln\left(\frac{y_{i(t-1)}}{y_{D(t-1)}}\right) + a_{i3} \cdot \ln\left(\frac{(\prod_{j=1, j\neq i}^{D-1} y_{j,t-1})^{(1/(D-2))}}{y_{D(t-1)}}\right) \tag{4}$$

Maximum likelihood estimation is carried out using the Nelder-Mead method in the `optim` function of R [10] to maximize the log-likelihood. However, in order to obtain the initial parameters of the optimization procedure, a ridge regression is considered (see details in [3]). The parameters are $\tau, a_{i1}, a_{i2}, a_{i3}$ for $i = 1, \ldots, D-1$. Note that working with equation (4) we can obtain the value of the Dirichlet parameters and calculate the expected value using $E[y_{it}] = \alpha_{it}/(\sum_1^D \alpha_{it})$.

The interpretation of the model parameters offers insights into both the individual contribution of each taxon and the influence of the broader microbial community on the temporal dynamics of bacterial abundances. Specifically, the parameter a_{i1} acts as an intercept and reflects the baseline abundance of taxon i. Higher values of a_{i1} are associated with consistently higher

abundances of the taxon across time points, independent of interactions with other taxa or its own past state, suggesting that the taxon tends to maintain a dominant or persistent presence in the microbial community. The parameter a_{i2} is associated with the additive log-ratio (alr) transformation of taxon i at time $t-1$, capturing the degree of temporal self-dependence. This coefficient quantifies how strongly the previous abundance of taxon i contributes to its own abundance at the subsequent time point. Biologically, a high value of a_{i2} indicates that the taxon follows a self-driven dynamic, potentially reflecting niche stability or low sensitivity to community-level fluctuations. In contrast, a_{i3} represents the influence of the rest of the microbial community on taxon i through a compositional balance. Specifically, this balance compares the aggregated abundance (in the alr-transformed space) of all taxa except taxon i and the most abundant taxon (placed in the numerator) against the most abundant taxon (in the denominator). The coefficient a_{i3} therefore quantifies how changes in the structure of the surrounding microbial community affect the future abundance of taxon i. A large magnitude of a_{i3} implies that taxon i is highly responsive to shifts in community composition, potentially pointing to ecological dependence, sensitivity to competition, or susceptibility to environmental changes mediated through other taxa.

• **BPBM**. We call the model defined in [4] BPBM. Let $\boldsymbol{y_t} = (y_{1t}, y_{2t}, \ldots, y_{Dt})$ where y_{it} denotes the relative abundance of the bacterial taxon i at time t where $i = 1, ..., D$. As in previous models, $\boldsymbol{y_t} \sim \text{Dirichlet}(\boldsymbol{\alpha_t})$, $\forall t$. This model proposes that:

$$\ln(\alpha_{it}) = \mu_{it} = a_{i0} + \sum_{j=1}^{M} a_{ij} \cdot \text{SPBal}_{j,t-1} \tag{5}$$

In this expression, M is the total amount of Selected Principal Balances (SPBal). The SPBal are the Principal Balances for which the sum of the percentage of variance is higher than 80% and they are obtained with Ward's clustering method [6]. See details in [8]. $\text{SPBal}_{j,t-1}$ denotes the selected principal balance j calculated using the relative abundance of the taxa at $t-1$. In addition, the Gaussian distribution with zero mean and standard deviation σ_{ij} is consider as the prior distribution of a_{ij}. As is suggested in [7], σ_{ij} is assigned the Uniform distribution in the interval $[0, 5]$. The parameters a_{ij} are interpreted as the weight that the relationship between the groups of taxa present in the SPBal_j has in defining the abundance of the taxon i at the next time point. The parameters a_{i0} are the intercepts. The value of the

SPBal_j gives information about the relationship between the groups in the numerator and denominator of the balance. Note that $\alpha_{it} = \exp(\mu_{it})$.

BPBM is defined considering the selected principal balances because we assume that balances with more variability will better explain the variability of the data. See details in [4]. Bayesian estimation is carried out using Markov Chain Monte Carlo (MCMC). This formulation enables the encapsulation of complex information within a compact structure, effectively reducing the number of parameters that need to be estimated. A direct consequence of this parameter reduction is the enhanced ability to model datasets that contain a higher number of microbial taxa, thereby improving scalability and facilitating the analysis of richly diverse microbial communities.

From a biological standpoint, the BPBM model provides a principled framework for understanding how large-scale structural shifts in microbial communities influence the temporal behavior of individual taxa. Central to this approach are the Selected Principal Balances (SPBals), which are log-ratio contrasts between groups of taxa chosen for their ability to explain the majority of variance in the compositional data. These balances reflect dominant ecological gradients within the community—such as contrasts between functionally distinct groups (e.g., fermenters vs. degraders), environmental niches (e.g., aerobic vs. anaerobic taxa), or health-related microbial assemblages (e.g., pathogenic vs. commensal taxa). By summarizing complex interactions into interpretable, orthogonal components, SPBals allow for the identification of key ecological axes that drive microbial community structure. Instead of modeling all pairwise taxon interactions—which becomes infeasible in high-dimensional settings—SPBals reduce the dimensionality of the problem while preserving ecological interpretability. This is particularly valuable when investigating how changes in the balance between taxonomic groups influence the future abundance of specific taxa.

In the BPBM framework, the intercept term a_{i0} captures the baseline abundance of taxon i, independent of community composition. Each coefficient a_{ij} quantifies the influence of the j-th SPBal at time $t-1$ on the abundance of taxon i at time t. A positive coefficient suggests that a relative increase in the numerator group of the balance promotes the growth or persistence of taxon i, while a negative value implies that such a shift has an inhibitory effect. Importantly, because SPBals aggregate taxa based on shared variance and ecological behavior, the model allows researchers to link the response of a focal taxon to broader functional or ecological group dynamics, rather than isolated pairwise relationships.

This biologically grounded use of SPBals enables the identification of taxa that are sensitive to specific community-wide patterns, revealing potential ecological dependencies, competition, or cooperative dynamics. The magnitude of a_{ij} reflects the degree of this sensitivity, with higher absolute values indicating a stronger association between taxon i and the ecological gradient captured by SPBal j. As a result, BPBM offers an interpretable, scalable approach to studying the drivers of taxon-specific dynamics within complex microbial ecosystems, making it particularly well-suited for high-dimensional microbiome time series data.

References

[1] J. Aitchison. *The statistical analysis of compositional data.* Chapman and Hall, 1986.

[2] I. Creus Marti, A. Moya, and F. J. Santonja. A statistical model with a lotka-volterra structure for microbiota data. 2018. Modelling for engineering and human behaviour 2018. Instituto Universitario de Matematica Multidisciplinar. ISBN: 978-84-09-07541-6.

[3] I. Creus Martí, A. Moya, and F. J. Santonja. A dirichlet autoregressive model for the analysis of microbiota time-series data. *Complexity, Hindawi*, 2021.

[4] I. Creus Martí, A. Moya, and F. J. Santonja. Bayesian hierarchical compositional models for analysing longitudinal abundance data from microbiome studies. *Complexity, Hindawi*, 2022.

[5] J. J. Egozcue and V. Pawlowsky-Glahn. Groups of parts and their balances in compositional data analysis. *Mathematical Geology*, 37:795–828, 2005. doi: 10.1007/s11004-005-7381-9.

[6] B. S. Everitt, S. Landau, M. Leese, and D. Stahl. *Cluster Analysis.* Wiley, Chichester, 2011.

[7] A. Gelman. Prior distributions for variance parameters in hierarchical models. *Bayesian Analysis*, 1:515–533, 2006.

[8] J. Martín-Fernández, V. Pawlowsky-Glahn, J.J. Egozcue, and R. Tolosona-Delgado. *Advances in Principal Balances for Compositional Data*. Mathematical Geosciences, 2018.

[9] V. Pawlowsky-Glahn and J. J Egozcue. Principal balances. *Proceedings of the 4th International Workshop on Compositional Data Analysis*, 2011.

[10] R Core Team. *R: A Language and Environment for Statistical Computing*. R Foundation for Statistical Computing, Vienna, Austria, 2021. URL `https://www.R-project.org/`.
