# Supplementary material for: CoDaLoMic: An R package for modeling microbiome compositional and longitudinal data
Source: PLoS Comput Biol. 2026 Jun 22;22(6):e1014328. doi: 10.1371/journal.pcbi.1014328 (PMC13362355; doi:10.1371/journal.pcbi.1014328)
Supplement: S1 Text — A document that includes the tables included in Figs 1 and 2 in an editable, cell‑based LATEX format. (PDF) [file pcbi.1014328.s017.pdf]

Supporting Information

Irene Creus-Martí[1,*], Andrés Moya[2, 3, 4], Francisco J. Santonja[1]

1 Department of Statistics and Operation Research, Universitat de València, Valencia, Spain
2 Institute for Integrative Systems Biology (I2Sysbio), Universitat de València and CSIC, València, Spain
3 The Foundation for the Promotion of Health and Biomedical Research of Valencia Region (FISABIO), Valencia, Spain
4 CIBER in Epidemiology and Public Health (CIBERESP), Madrid, Spain

* irene.creus@uv.es

1 Supporting Information

We provide the tables originally included in Figure 1 and Figure 2 of the manuscript in an editable, cell-based LaTeX format. The tables corresponding to Figures 1B, 1C, and 1F are Tables A, B, and C, respectively. The table corresponding to Figure 2H is Table D.

Table A: Estimated parameters obtained with the Dirich-gLV model using the simulated dataset.

Names	Weight that the bacteria has in defining herself in the next time point	Weight that the interaction of both bacteria has in defining the bacteria in in the row in the next time point			
		sp1	sp2	sp3	sp4
sp1	-0.67	-1.51	0.86	0.78	0.41
sp2	-1.42	0.68	-0.56	0.29	0.02
sp3	-0.05	-0.57	-0.77	2.4	-0.34
sp4	0.58	0.78	1.1	-2.85	-0.08

Table B: Estimated parameters obtained with the FBM model using the simulated dataset.

Name	Intercept	Weight of the bacteria in defining herself in the next time point	Weight of the rest of the community in defining the bacteria in the next time point
sp1	0.44	-0.55	0.35
sp2	0.07	-0.23	0.07
sp3	0.47	-0.89	0.35
sp4	-0.07	0.34	-0.70

Table C: Estimated parameters obtained with the BPBM model using the simulated dataset. We rename each bacteria with a number so 1 is sp1, 2 is sp2, 3 is sp3, 4 is sp4, 5 is sp5.

SPBal (% of variance)	NUM/DEM	Bacteria in NUM/DEM	SPBal mean (Relation between NUM/DEM)	Genera most influenced by the SPBal
SPBal1<br>(50.54%)	NUM<br>DEM	1,3,2,5<br>4	0.389<br>(Similar)	2
SPBal2<br>(26.8%)	NUM<br>DEM	1,3,2<br>5	0.125<br>(Similar)	
SPBal3<br>(18.24%)	NUM<br>DEM	1,3<br>2	0.145<br>(Similar)	1,2,4

Table D: Mean values of the quality metrics obtained with the Dirich-gLV, FBM, and BPBM models.

	Data used to estimate				Data used to predict				Entire Dataset			
	RMSD	NSC	RSS	MAPE	RMSD	NSC	RSS	MAPE	RMSD	NSC	RSS	MAPE
Dirich-gLV	0.08	0.16	0.06	0.95	0.13	-14.33	0.04	1.25	0.10	-0.16	0.10	1.02
FBM	0.09	-0.08	0.06	1.65	0.10	-4.67	0.03	1.17	0.10	-0.05	0.09	1.55
BPBM	0.07	0.32	0.04	1.23	0.10	-6.66	0.02	1.26	0.08	0.23	0.06	1.24
